# A multi-parametric screening platform for photosynthetic trait characterization of microalgae and cyanobacteria under inorganic carbon limitation

**DOI:** 10.1371/journal.pone.0236188

**Published:** 2020-07-23

**Authors:** Priyanka Pradeep Patil, Imre Vass, Sandeesha Kodru, Milán Szabó

**Affiliations:** 1 Institute of Plant Biology, Biological Research Centre, Szeged, Hungary; 2 Biology PhD School, Faculty of Science and Informatics, University of Szeged, Szeged, Hungary; 3 Climate Change Cluster, University of Technology Sydney, Ultimo, Australia; National Research Council of Italy, ITALY

## Abstract

Microalgae and cyanobacteria are considered as important model organisms to investigate the biology of photosynthesis; moreover, they are valuable sources of biomolecules for several biotechnological applications. Understanding the species-specific traits of photosynthetic electron transport is extremely important, because it contributes to the regulation of ATP/NADPH ratio, which has direct/indirect links to carbon fixation and other metabolic pathways and thus overall growth and biomass production. In the present work, a cuvette-based setup is developed, in which a combination of measurements of dissolved oxygen, pH, chlorophyll fluorescence and NADPH kinetics can be performed without disturbing the physiological status of the sample. The suitability of the system is demonstrated using a model cyanobacterium *Synechocystis* sp. PCC6803, as well as biofuel-candidate microalgae species, such as *Chlorella sorokiniana*, *Dunaliella salina* and *Nannochloropsis limnetica* undergoing inorganic carbon (Ci) limitation. Inorganic carbon limitation, induced by photosynthetic Ci uptake under continuous illumination, caused a decrease in the effective quantum yield of PSII (Y(II)) and loss of oxygen-evolving capacity in all species investigated here; these effects were largely recovered by the addition of NaHCO_3_. Detailed analysis of the dark-light and light-dark transitions of NADPH production/uptake and changes in chlorophyll fluorescence kinetics revealed species- and condition-specific responses. These responses indicate that the impact of decreased Calvin-Benson cycle activity on photosynthetic electron transport pathways involving several sections of the electron transport chain (such as electron transfer via the Q_A_-Q_B_-plastoquinone pool, the redox state of the plastoquinone pool) can be analyzed with high sensitivity in a comparative manner. Therefore, the integrated system presented here can be applied for screening for specific traits in several significant species at different stages of inorganic carbon limitation, a condition that strongly impacts primary productivity.

## Introduction

Microalgae are phototrophic organisms that play an essential role in the biogeochemical cycling of macro and microelements and are thus key components of freshwater and marine ecosystems. Besides their role in the biosphere, microalgae are also valuable sources of various compounds that have a great importance for human consumption, such as pharmaceuticals, nutraceuticals, food supplements etc. However, in order to efficiently utilize microalgae, it is essential to understand their physiology, life cycle and stress tolerance mechanisms in detail. Microalgae, as other oxygenic phototrophs, are often exposed to fluctuating environmental conditions, which can cause imbalances in metabolic reactions that ultimately affect growth and thus biomass production. The light reactions of photosynthesis, performed by several components of the photosynthetic electron transport chain, including Photosystem II (PSII), Cytochrome b_6_/f, Plastocyanin, Photosystem I (PSI), together with the subsequent carbon fixation processes provide rapid regulatory mechanisms that can fine-tune the energetic balance to mitigate the detrimental effects of highly variable stress conditions. Linear electron transport originating from the oxidation of water molecules produces ATP and NADPH, which are utilized primarily in the Calvin-Benson cycle. ATP and NADPH are produced in a ratio that is often insufficient for CO_2_ fixation [1]. Various stress factors can modify the ATP and NADPH generation pathways and thus the ATP/NADPH ratio, potentially causing imbalances in the cellular energy stocks. Stress conditions prime metabolism for elevated ATP demand, which can be provided by alternative electron transport mechanisms (reviewed e.g. in [2]). Cyclic electron flow around PSI (CEF) is exclusively involved in ATP synthesis without the accumulation of NADPH. Briefly, CEF involves transferring electrons from PSI to the PQ pool, mediated by the NAD(P)H PQ oxidoreductase pathway, namely the type 1 NADH dehydrogenase (NDH-1) or the type 2 NADPH dehydrogenase (NDH-2), or by the Proton Gradient Regulation/PGR5-Like Photosynthetic Phenotype 1 (PGR5/PGRL1) pathway (reviewed e.g. in [3]). This cyclic electron transfer mechanism is coupled with proton translocation from the stroma to the lumen during the Q-cycle [4], and the translocated protons are utilized by ATP synthase to produce ATP without the net synthesis of NADPH.

Inorganic carbon (Ci) in the form of bicarbonate (HCO_3_^-^) and/or carbon dioxide (CO_2_) is the most important and essential macronutrient for the growth of organisms performing oxygenic photosynthesis. Although inorganic carbon is crucial to sustain photosynthetic carbohydrate synthesis, aquatic photosynthetic organisms are often exposed to randomly fluctuating Ci availability or sustained Ci limitation that may strongly impact primary productivity and growth. To overcome Ci limitation, photosynthetic organisms operate carbon concentrating mechanisms (CCM) that play an important role in the physiological gas-exchange characteristics by increasing the concentration of CO_2_ at the active site of ribulose-1,5-bisphosphate carboxylase oxygenase (Rubisco) [5–8].

In previous works the activation of alternative electron transport mechanisms including CEF was characterized in cyanobacteria using various non-invasive biophysical methods. Variable chlorophyll fluorescence is a frequently applied tool to monitor the operating efficiency of PSII (e.g. [9, 10]), as chlorophyll fluorescence is relatively easily measured in standard cuvette systems as well as in photobioreactors, even in large scale algal cultivation facilities. The influence of inorganic carbon content on photosynthetic performance can be sensitively monitored by the Chlorophyll (Chl) fluorescence induction technique in green algae [11, 12] as well as in cyanobacteria [13, 14]. Advanced Chl fluorescence techniques such as post-illumination rise in chlorophyll fluorescence and flash-induced fluorescence decay kinetics [15] can also reveal the electron transfer steps between the Q_A_, Q_B_ and the PQ pool. In addition, a wave phenomenon observed in fluorescence decay was found to be particularly useful to resolve specific redox kinetics that reveal the operation of the PQ pool and electron donations towards PSI during CEF in cyanobacteria [15] and green algae [16]. Combination of Chl fluorescence with P700 kinetics, NADPH fluorescence and/or transient measurements of carotenoid bandshifts (electrochromic absorbance changes) have been previously applied to monitor the balance and shifts between LEF and CEF mechanisms under altered Ci conditions [13, 14, 17–19]. A thin-layer microcell capable of measuring flash O_2_ yields and chlorophyll fluorescence by fast repetition rate (FRR) fluorometry allowed the characterization of linear and cyclic electron flow within PSII in several microalgae and cyanobacteria [20] and the analysis of PSII binding sites of HCO_3_^-^ with different affinities under reversible inorganic carbon limitation [21].

The responses of photosynthetic electron transport to Ci limitation have been characterized in popular model species and mutants of cyanobacteria, e.g. *Synechocystis* PCC 6803 and green algae such as *Chlamydomonas reinhardtii* [6, 13, 17]. A particularly important and extensively characterized mutant of *Synechocystis* PCC 6803 is the ndhB-deficient M55 mutant, which lacks all the type 1 NADPH-dehydrogenase (NDH-1) complexes [22], and is therefore unable to perform CEF and CCM. The M55 has a high-CO_2_-requiring phenotype, its photosynthetic activity remains low, and the NADP pool is highly reduced under ambient CO_2_-concentrations [22–24].

The inherent regulation of photosynthesis and the plasticity of the photosynthetic electron transport chain under variable growth and environmental conditions and abiotic stress factors remains largely uncharacterized in several microalgae species that have high relevance in biotechnological applications and biofuel industries, such as *Chlorella sorokiniana*, *Dunaliella salina and Nannochloropsis limnetica*. The green alga *Chlorella sorokiniana* is considered as a stress-resilient species, and due to its ability to maintain high growth rates under stress it is applied in several industrial processes such as wastewater treatment (e.g. [25]) and elevated CO_2_ sequestration which enhances lipid production [26]. The species *Dunaliella salina*, a highly salt-tolerant green alga is applied for commercial scale *β*-carotene production in pharmaceutical and nutraceutical industries (recently reviewed in [27]). Furthermore, this species is also an important model organism for studying photoacclimation capabilities to enhance biomass productivity (e.g. [28]).

*Nannochloropsis limnetica* (Eustigmatophyceae) is a freshwater alga, whose total fatty acid content can be very high, even double of the fatty acid content of other marine *Nannochloropsis* species [29]. Despite the high relevance of these species in industrial applications, the dynamic regulation of their photosynthetic responses, e.g. under transient Ci limitation has scarcely been characterized.

Precisely designed and operated photobioreactor systems with multiple sensors of physico-chemical parameters (dissolved oxygen, pH, optical density) and gas control allowed real time monitoring of the physiological changes and net photosynthesis under Ci limitation [30–33]. However, an integrated analysis system, where the physico-chemical parameters of algae suspensions can be measured along with photosynthetic efficiency parameters and the kinetics of photosynthetic electron transport in a simple cuvette-based platform has not been established and utilized for screening for photosynthetic markers in microalgae. A standardized, cuvette-based platform that allows monitoring dissolved O_2_ and pH along with non-invasive measurements of advanced Chl fluorescence and NADPH fluorescence kinetics would allow screening for photosynthetic traits of uncharacterized species, under various stages of Ci limitation and recovery.

Therefore the aims of the present work were (i) to establish an integrated cuvette system, where several parameters (O_2_ evolution/uptake, pH) and photosynthetic quantum efficiency and kinetics of photosynthetic electron transport can be simultaneously recorded and monitored, in time-resolved manner under well-defined environmental conditions, and (ii) to comprehensively and comparatively monitor and assess the photo-physiological and photosynthetic behavior of *Synechocystis* sp. *PCC6803 WT* (grown with and without CO_2_), the M55 mutant of *Synechocystis* sp. *PCC6803*, *Chlorella sorokiniana*, *Dunaliella salina and Nannochloropsis limnetica* under inorganic carbon limitation and during recovery from inorganic carbon limitation stress. This integrated approach would allow to investigate photosynthetic efficiency and operation of photosynthetic electron transport pathways in biofuel-candidate microalgae undergoing inorganic carbon limitation, a particularly relevant stress condition that affects photosynthetic performance and primary productivity in short timescales.

## Materials and methods

### Culture conditions

*Synechocystis* sp. PCC 6803 WT (wild type) cells were grown in BG-11 medium on a rotary shaker under continuous illumination of 40 μmol photons m^-2^ s^-1^ photon flux density white light at 30°C, supplied by 3% CO_2_ or ambient CO_2_ (where ‘ambient’ refers to approx. 410 ppm atmospheric CO_2_ concentration). The M55 mutant (ΔndhB), deficient in type I NADPH dehydrogenase complex, NDH-1 [22] of *Synechocystis* was grown under the same conditions as the WT (in 3% CO_2_) in BG-11 medium supplemented with kanamycin (20 μg mL^−1^).

*Chlorella sorokiniana* (Trebouxiophyceae) and *Nannochloropsis limnetica* (Eustigmatophyceae) were grown in 500 mL Erlenmeyer flasks with 200 mL culture volume in BG-11 medium with the light intensity of 56 μmol photons m^-2^ s^-1^ (white LED light), 12 h: 12 h day: night diurnal cycle with continuous shaking at 120 rpm, at a constant temperature of 24°C.

*Dunaliella salina* was grown under the same conditions as described above, but in f/2 medium (prepared with artificial seawater) supplemented with 30 gL^-1^ NaCl and no shaking.

For measurements the cells were harvested in the early exponential growth phase, centrifuged at 6,500 g for 5 min at 24°C and were resuspended in fresh medium to achieve the Chl *a* content of 5 μg mL^-1^.

### Measurement of chlorophyll content

For eukaryotic microalgae chlorophyll was determined using acetone and DMSO (1:1). The samples were centrifuged at 16,000 g for 8 min. The supernatant was discarded and 1 mL of a 1:1 mixture of 90% acetone and 90% DMSO was added to the pellet, then incubated on ice for 5 min, vortexed and centrifuged at 16,000 g for 7 min. The supernatant containing the extracted pigments was transferred to a glass cuvette with 1 cm path length for measurement. The absorbance spectrum was measured with a UV-visible spectrophotometer (UV-1601, Shimadzu corporation, Japan) at A_663_ and A_645_ nm for determining the chlorophyll *a* content, according to [34, 35] (see also S1 Table). Eq 1 was used for calculating chlorophyll *a* content for microalgae.

$$Chl\;a\;\left(\mu g\;mL^{‐1}\right)=11.75*A_{663}‐2.35*A_{645}$$ (1)

For cyanobacteria, the chlorophyll *a* content was determined by 100% methanol extraction. Eq 2 was used to calculate chlorophyll *a* content for *Synechocystis* sp.

$$Chl\;a\;\left(\mu g\;mL^{‐1}\right)=16.5*A_{665.5}‐8.3*A_{650.5}$$ (2)

### Experimental setup

**Fig 1:**
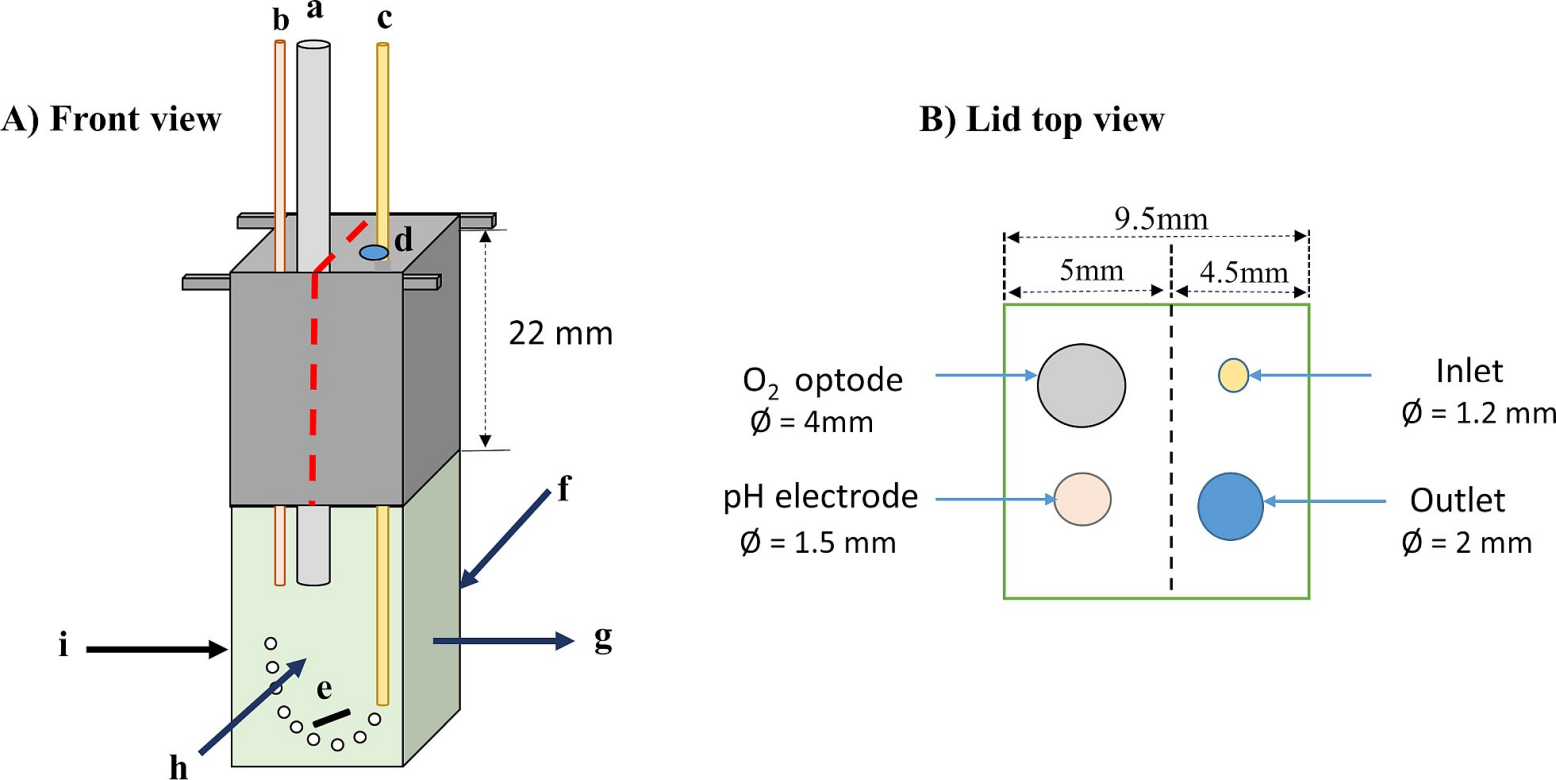
Cuvette-based setup with custom-designed lid including (a) O_2_ optode, (b) pH electrode, (c) Inlet (air/gas bubbling), (d) Outlet, (e) Magnetic stirrer, (f) NADPH emitter, (g) NADPH detector, (h) Chlorophyll fluorescence detector, (i) Fiber optic white light source.

A cuvette-based sample holder (Optical Unit ED-101US/MD, Heinz Walz GmbH, Effeltrich, Germany) attached to a DUAL-PAM-100 chlorophyll fluorometer (Heinz Walz GmbH, Effeltrich, Germany) was used for monitoring the physiological response of microalgae. A quartz cuvette with 1 cm path length (Hellma GmbH, Germany) was used with a custom-designed lid, in which various probes were fixed in the following configuration (as shown in Fig 1): (a) a 4 mm outside diameter O_2_ optode (Fibox 3, Presens GmbH, Aachen, Germany) to measure the dissolved oxygen content of the sample, (b) a 1.5 mm pH electrode (Microelectrode Inc., USA) to log the pH change, (c) a 1.2 mm inlet pipe to provide air bubbling controlled via a 18-gauge blunt tip syringe needle to avoid the buildup of excess oxygen concentration that may cause oxygen toxicity in the chamber, and (d) an outlet hole for gas exchange and pressure release.

The lid was designed in two parts: half of the lid could be easily opened and closed when needed during the experiment without disturbing the O_2_ optode and the pH electrode, which can stay stable together with the other half of the lid. The temperature was maintained at 24°C for eukaryotic microalgae and 30°C for cyanobacteria in a temperature-controlled block (ED-101US/T Heinz Walz GmbH, Effeltrich, Germany) fixed on the Optical Unit ED-101US/MD, by using a water circulation heater chiller (Pharmacia Biotech GmbH MultiTemp III, Germany). The cuvette was closed for the duration of the O_2_ evolution measurement, and opened after the measurement to allow gas exchange and to avoid the buildup of excess levels of dissolved oxygen that may cause oxygen toxicity (according to [12]). The culture was continuously stirred with a small magnetic stirrer bar. The sample was illuminated with white actinic light provided by a fiber optic light source from one side of the cuvette (Schott KL 1500, Schott AG, Germany). For NADPH measurements the Dual NADPH/9AA module was used (see below), with the emitter (Dual-ENADPH) and detector (Dual-DNAPH) units placed at right angle position to avoid the measuring light reaching the photomultiplier and reducing the level of artificial background signals. Chlorophyll fluorescence was recorded with a Dual-DB/DR unit, assembled to a right angle position to the Dual-DNADPH detector (Fig 1). Flash-induced fluorescence decay kinetics and OJIP transient curves were measured using a double-modulation PSI fluorometer (see below) that was not directly integrated into the Dual-PAM cuvette unit. The PSI-FL3000 fluorometer was equipped with a fixed measuring head (the so-called super-head, containing the cuvette holder and the emitter detector unit), therefore the cuvette was transferred from the Dual-PAM holder into the PSI super-head.

### Experimental procedure

**Fig 2:**
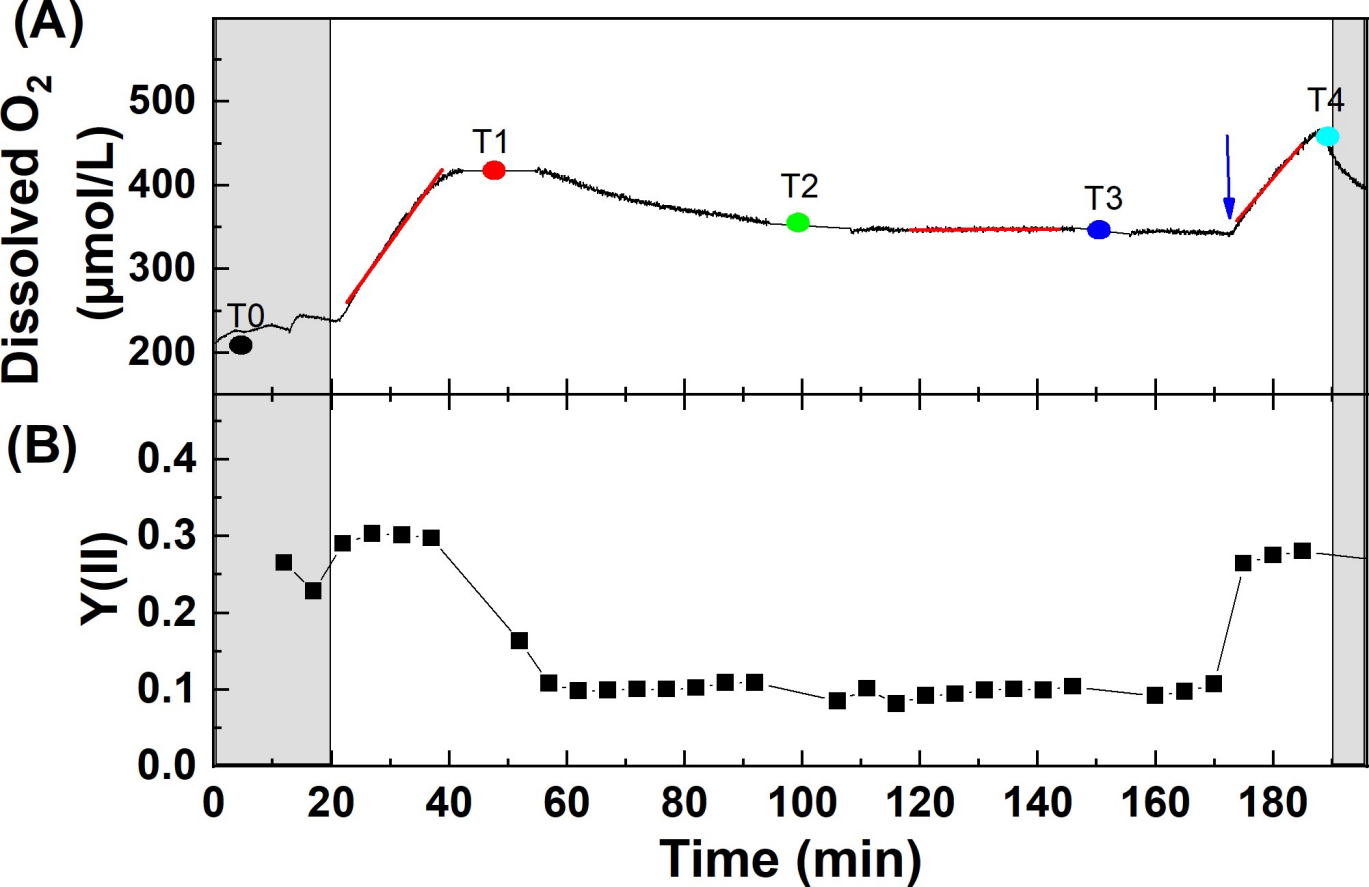
Representative graph of the physiological response of *Synechocystis* sp. PCC6803 WT during the course of Ci limitation. The cells were washed and resuspended in fresh HEPES buffered BG11 medium. Simultaneous measurements of (A) Oxygen evolution and (B) Effective quantum yield of PSII (Y(II)) were performed. Y(II) was measured every 5 min. The cells were kept in the dark for 20 min (grey shade region), then illuminated with actinic white light at 56 μmol photons m^-2^ s^-1^. The dots in panel A represent the defined measuring time points of chlorophyll and NADPH fluorescence kinetics at: T0 (control) in the dark, T1—initial Ci-replete phase in the light, T2—early phase of Ci depletion, T3—prolonged Ci-depleted phase, T4—Ci recovery phase in the light (arrow indicates the addition of 10 mM NaHCO_3_). The red lines represent the phases to determine O_2_ evolution/uptake rates.

The photophysiological response of microalgae was investigated by using variable chlorophyll fluorescence via pulse amplitude modulation (PAM) chlorophyll fluorometry, O_2_ evolution and pH measurements. 2 mL working volume was loaded in the 1-cm rectangular quartz cuvette with 5 μg mL^-1^ Chl *a* suspended in fresh medium for the measurement. The cuvette was covered with a customized lid ensuring that the sample was closed, or open to gas exchange with ambient air, depending on the applied experimental conditions. The dissolved oxygen concentration was logged continuously, measured at a sampling interval of every 1 s by the manufacturer’s software (OxyView—PST3-V5.32 02/2004, PreSens GmbH, Germany) via USB connection. The O_2_ sensor was calibrated with two points, i.e. air-saturated water ‘Cal 100’ (100% air saturation) and anoxic water ‘Cal 0’ (1 g of Na_2_SO_3_ in 100 ml distilled water) before the measurement. The oxygen evolution/uptake rate was determined from the slope of the linear fitting of the original traces and expressed in μmol O_2_ [mg Chl *a*]^-1^ h^-1^ units. In certain experiments, changes in O_2_ evolution/uptake dynamics are displayed as a rate, given by the first derivative of the dissolved oxygen traces. pH data were logged every 5 min via the manufacturer’s data logging software (Orion Star A300, Thermo Scientific Inc., USA). Effective quantum yield, oxygen concentration and pH were recorded starting from the 0 min time point. In the first 20–30 min the sample was kept in the dark with a weak measuring light on to determine the minimum fluorescence (denoted as F_0_ in the dark-adapted state or F_s_ in the light-adapted state). A 600 ms saturation light pulse (LED with peak emission at 635 nm, PPFD 10,000 μmol photons m^-2^ s^-1^) was applied every 5 min for the determination of maximum fluorescence (denoted as F_m_ in the dark-adapted state or F_m_’ in the light-adapted state). The effective quantum yield of photochemical energy conversion in PSII in the light-adapted state was calculated as Y(II) = (F_m_^’^–F_s_)/F_m_^’^ or in analogy, the expression F_v_/F_m_ = (F_m_−F_0_)/F_m_ was applied to determine the maximum quantum efficiency of PSII in the dark-adapted state [36]. After a dark period of approx. 25 min the sample was illuminated with actinic white light with the same irradiance that was applied as a growth light, to induce inorganic carbon uptake, until cells achieved a Ci-limited state. Each species required different time periods to reach the Ci-depleted phase, therefore the AL was provided for specified time periods as indicated at the individual experiments (see below in Fig 2 and S1–S6 Figs). To restore the photosynthetic activity of the cells, 10 mM NaHCO_3_ was added at the fully Ci-depleted stage. At defined time points, the NADPH and Chl fluorescence measurements were performed as specified below. The flash-induced fluorescence decay kinetics and OJIP transient curves were measured by transferring the cuvette to the PSI measuring head without removing the lid or the sensors, therefore neither the physiological and physico-chemical conditions of the cell suspensions were disturbed, nor the Ci content of the cells and the geometry of the cuvette setup were changed throughout the whole process/experiment.

### Simultaneous measurement of NADPH and chlorophyll fluorescence

NADPH and chlorophyll fluorescence were simultaneously measured in a Dual-PAM-100 chlorophyll fluorometer (Heinz Walz, Effeltrich, Germany) at 24°C for green algae and 30°C for cyanobacteria. At a specific time point representing either the Ci-replete or -depleted stage (defined as T points, see Results and Discussion), cells were dark-adapted for 3 min. A slow induction measuring protocol was initiated and the NADPH/Chl fluorescence signals were recorded for 30 s in the dark to establish a baseline, then the actinic red light (635 nm peak intensity) at ~56 μmol photons m^-2^ s^-1^ (or near the growth light intensity) was switched on to record light-induced NADPH/Chl fluorescence change for 180 s, and subsequently the NADPH/Chl fluorescence change from light-to-dark was monitored up to 300 s.

NADPH fluorescence was measured using the NADPH/9-AA module of a Dual-PAM-100 instrument (Heinz Walz, Effeltrich, Germany; [37–39]). The NADPH/9-AA module consists of an emitter unit (DUAL-ENADPH) and a detector unit (DUAL-DNADPH). NADPH fluorescence was excited by UV-A (365 nm) from the DUAL-ENADPH unit and detected by a blue-sensitive photomultiplier with a filter transmitting light between 420 and 580 nm in the DUAL-DNADPH unit. The measuring light intensity was set to 20, the measuring light frequency in the absence and presence of red AL was set at 200 and 5,000 Hz, respectively. Chl fluorescence was detected with a Dual-DB/DR unit (see Fig 1 for the topological arrangement of the measuring setup and refer to [38] for the technical details of the emitter-detector units).

### Measurement of flash-induced chlorophyll fluorescence decay and OJIP transients

Flash-induced chlorophyll fluorescence decay was measured in the same cuvette that was used for measuring simultaneous Chl-NADPH fluorescence using a double-modulation fluorometer (FL-3000, Photon System Instruments, Drásov, Czech Republic). The measuring unit was controlled by the manufacturer’s software (FluorWin, Photon System Instruments, Drásov, Czech Republic). The timing and duration of the flashes were defined in the FluorWin experimental protocol and executed by a microprocessor in the control unit of the fluorometer. After 4 measuring flashes (8 μs, 620 nm) to determine minimum fluorescence in the dark, a single turnover saturating actinic flash (30 μs, 639 nm) was applied, which transfers an electron from the water oxidizing complex to Q_A_, resulting in a prompt increase in fluorescence as a result of formation of Q_A_^-^ [15]. The fluorescence decay resulting from reoxidation of Q_A_^-^ was measured by applying measuring flashes in the time range from 150 μs to 100 s on a logarithmic time scale. OJIP transient curves were recorded by applying continuous illumination with strong actinic light for 3 s (639 nm peak wavelength, PPFD ~3,000 μmol photons m^-2^ s^-1^).

### Data analysis

Graphs were plotted and analyzed using OriginPro 2018 software. To test statistical differences between the Ci replete, depleted and recovery, paired-sample t-test was applied using the OriginPro 2018 software. The flash-induced fluorescence decay curves and the OJIP transients were double normalized; minimal fluorescence F_0_ was set to 0 and maximal fluorescence was set to 1.

## Results and discussion

### Continuous monitoring of physiological changes under Ci limitation

In a typical experiment in WT *Synechocystis* cells (Fig 2), after recording the respiratory oxygen uptake in the dark, a significant increase in dissolved oxygen content was observed in the presence of actinic light, indicating active oxygen evolution by photosynthesis. Upon prolonged illumination, the rate of oxygen evolution decreased as cells gradually entered a Ci-depleted stage. After about 20 min of illumination, oxygen evolving capacity was reversibly lost as *Synechocystis* cells became Ci-depleted, and the dissolved oxygen level slightly decreased as a result of oxygen uptake by the cells, potentially due to the activation of oxygen uptake mechanisms (e.g. [40]). The effective quantum yield of PSII (Y(II)) in the light-adapted state (or F_v_/F_m_, maximum quantum yield of PSII in the dark-adapted state) increased upon illumination (during dark to light transition), probably due to state 2 to state 1 transition [41], then after attaining a plateau for 15–20 min, Y(II) decreased gradually during the course of prolonged illumination and attained a steady-state level after ~25 min of illumination (Fig 2). After addition of NaHCO_3_, Y(II) and oxygen evolution recovered to 80–90% of the values observed at the beginning of the illumination (initially carbon-replete stage), indicating that the decrease in oxygen evolution capacity and effective quantum yield occurred as a result of Ci depletion during illumination (the irradiance was unchanged during NaHCO_3_ addition). The results of continuous monitoring of dissolved O_2_, Y(II), and pH for the entire experiments for all species are shown in the Supplementary material (S1–S6 Figs). The pH during the experiments remained unchanged in the cultures grown and suspended in BG-11 (pH = 7.5–7.7, S1–S5 Figs). However, in the case of *D*. *salina* cultivated in f/2 medium, the pH showed a progressive increase from pH = 7.7 to a plateau of 9.2 (S6 Fig), as the cells entered the Ci-limited stage (for the relationship between extracellular pH and Ci content in algae cultivated in an artificial seawater based medium, refer to [18, 32]). The simultaneously recorded oxygen and chlorophyll fluorescence data in the integrated cuvette system allows displaying the dynamic changes in photosynthetic processes in various stages of Ci limitation/recovery. Dynamic changes in oxygen evolving capacity (i.e. the rate, given by the first derivative of changes in dissolved oxygen level) displayed along with PSII electron transfer rate (ETR) showed a good correlation in all species. However, in some cases at the beginning of the experiment, especially in microalgae, oxygen evolution rate was found to be relatively low as compared to Y(II), which is possibly due to the operation of active oxygen uptake mechanisms (S7 Fig shows the original plots for all species investigated here).

The reversible loss of O_2_ evolution capacity and decrease in PSII quantum yield under bicarbonate depletion are in agreement with earlier findings that were obtained by simultaneous O_2_ yield and variable Chl fluorescence assays. In Ref. [42] it was found that addition of 10 mM NaHCO_3_ to Ci-depleted *Synechocystis* cells caused a 2-fold stimulation of oxygen evolution. In a mutant that is deficient of the arginine binding site of the CP43 protein (R357S), the stimulating effect of NaHCO_3_ addition on O_2_ evolution was much less expressed or completely missing. Bicarbonate has an important regulatory role in both the donor and acceptor sides of PSII (reviewed e.g. in [43]). On the donor side, bicarbonate was found to play a role in photo-induced assembly of the manganese cluster of the water-oxidizing complex, stabilization of PSII and protection of PSII against photoinhibition and thermoinactivation (reviewed in [44]). On the acceptor side bicarbonate is coordinated to the non-heme iron located between Q_A_ and Q_B_ [45] and therefore plays an important role in the regulation of electron transport from Q_A_^-^ to Q_B_, the protonation of Q_B_^2-^ and the exchange of Q_B_H_2_ with the PQ pool (studied extensively by Govindjee and colleagues, reviewed e.g. in [46, 47]).

### Comparative analysis of effective quantum yield of PSII under Ci limitation

**Fig 3:**
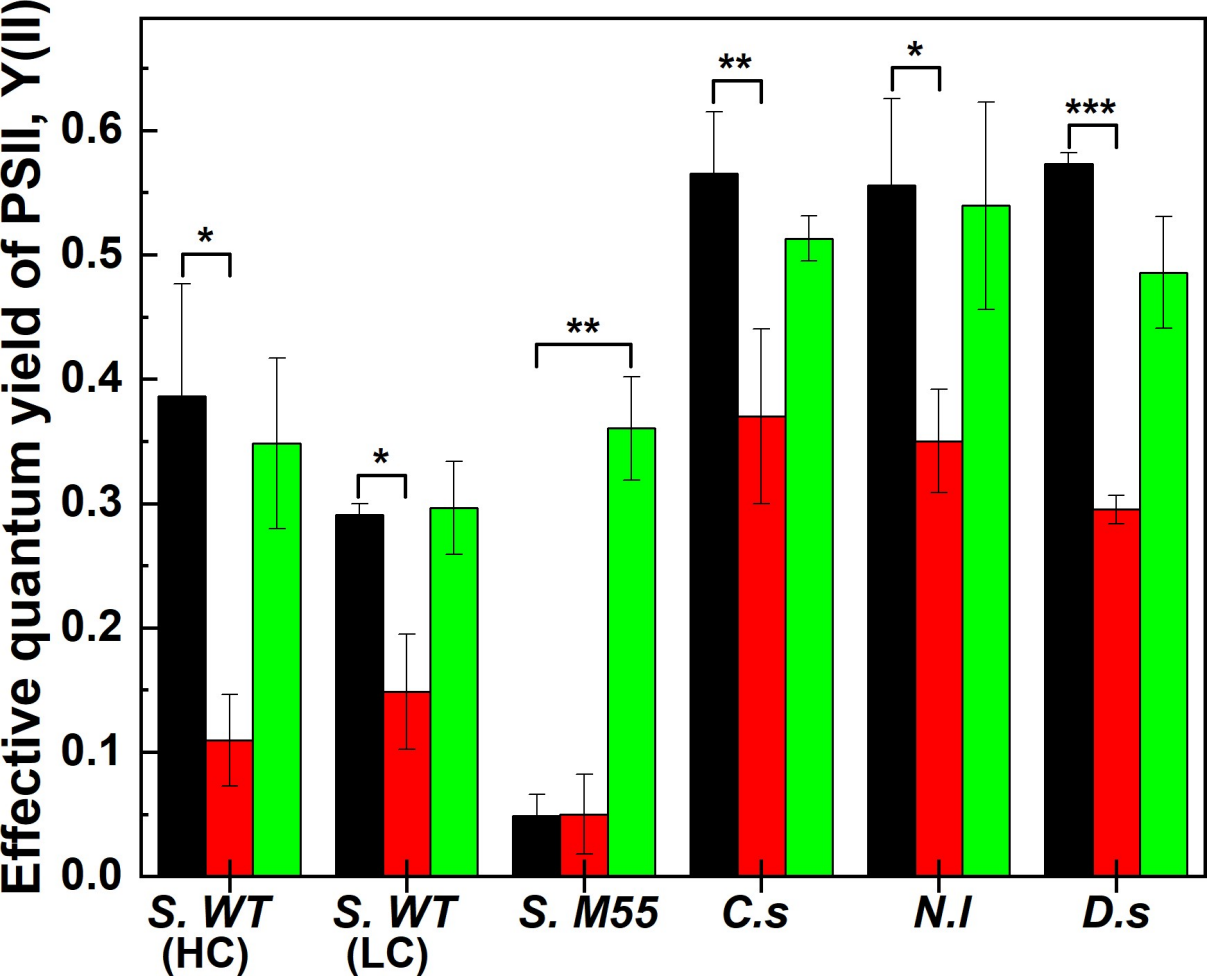
**Effective quantum yield of PSII in Ci-replete (black), -depleted (red) and recovery (green) stages.** The values are presented as a mean ± SD (n = 3) indicated by error bars. The asterisks mark significant difference between the indicated states (single asterisks (*): p<0.05, double asterisks (**): p<0.01, triple asterisks (***): p<0.001). Abbreviations: S. WT (HC) (*Synechocystis* sp. PCC6803 WT High CO_2_ grown), S. WT (LC) (*Synechocystis* sp. PCC6803 WT Low (ambient) CO_2_ grown), S. M55 (*Synechocystis* M55 mutant), C.s (*Chlorella sorokiniana*), N.l (*Nannochloropsis limnetica*), D.s (*Dunaliella salina*).

Effective quantum yield of PSII (Y(II)) in the initial Ci replete stage (T1 phase) showed specific differences in *Synechocystis* sp. PCC6803; cultures grown in the presence of 3% CO_2_ and at ambient CO_2_ exhibited Y(II) = 0.39±0.1 and Y(II) = 0.29±0.01, respectively, whereas the M55 mutant showed Y(II) = 0.05±0.02. Under prolonged Ci-limited stage (T3 phase), Y(II) significantly dropped to 0.11±0.04 and to 0.15±0.05 in cells grown at 3% CO_2_ and at ambient CO_2_, respectively (when compared to Ci-replete, p<0.05, Fig 3), while in the M55 mutant Y(II) remained essentially unchanged (Y(II) = 0.05±0.03). Upon addition of NaHCO_3_, Y(II) recovered to 0.35±0.07 and to 0.29±0.04 in cultures grown at 3% CO_2_ and at ambient CO_2_, respectively, and to 0.36±0.04 in the M55 mutant. This indicates that the M55 cells were probably Ci-limited from the beginning (even though they were cultivated at 3% CO_2_), and only after the addition of 10 mM NaHCO_3_ did they regain maximal PSII quantum efficiency. This was not due to lack of functional PSII reaction centres, because dark-adapted F_v_/F_m_ was found to be 0.45±0.02 in the M55 mutant (S3 Fig).

In the species *C*. *sorokiniana*, *D*. *salina* and *N*. *limnetica*, the behavior of Y(II) under Ci depletion and recovery was similar. With the continuous illumination of actinic white light (for approx. 3.5h), Y(II) significantly decreased, and upon addition of NaHCO_3_ Y(II) recovered nearly completely in all three species. The dynamics of the chlorophyll fluorescence parameters (original chlorophyll fluorescence traces are shown in S8 Fig) indicates that minimal steady-state fluorescence (F_s_) in *Synechocystis* grown at 3% CO_2_ increases during actinic illumination and remains at a high level during the onset of Ci limitation, and after addition of NaHCO_3_ it recovers to the initial Ci-replete stage (S8A Fig). However, *Synechocystis* grown at ambient CO_2_ exhibited only a small increase in F_s_ under actinic illumination (S8B Fig), suggesting that the PQ pool remained at a more oxidized state during the Ci limitation experiment in *Synechocystis* cells grown at ambient CO_2_ level as compared to the 3% CO_2_ growth condition. This effect is probably due to the operation of an alternative electron flow, such as photorespiration [48] (or more likely due to the flavodiiron-mediated O_2_ consumption [49]). M55 showed a rapid increase in steady-state fluorescence (F_s_) (S8C Fig) and a decrease in F_m_’ within 5 min of actinic light illumination that corresponds to the immediate drop in Y(II) in the light (S3 Fig). F_s_ remained at high level and F_m_’ remained smaller during the onset of Ci limitation, which was recovered after the addition of NaHCO_3_ to the initial Ci-replete stage. C. *sorokiniana* (S8D Fig) exhibited a moderate increase in F_s_ after 60–70 min of actinic illumination, which coincided with the cessation of oxygen evolution and drop in Y(II). Prolonged illumination caused a decrease in F_m_’, most probably due to the activation of non-photochemical quenching processes as a potential energy dissipating mechanism under Ci limitation. *N*. *limnetica* (S8E Fig) also exhibited an increase in F_s_ under prolonged illumination, however F_m_’ remained relatively constant under Ci limitation. *D*. *salina* (S8F Fig) exhibited a continuous and progressive decrease in both F_s_ and F_m_’ probably as a result of activation of non-photochemical quenching mechanisms under Ci limitation.

### Comparative analysis of photosynthetic oxygen evolution rates under Ci limitation

**Fig 4:**
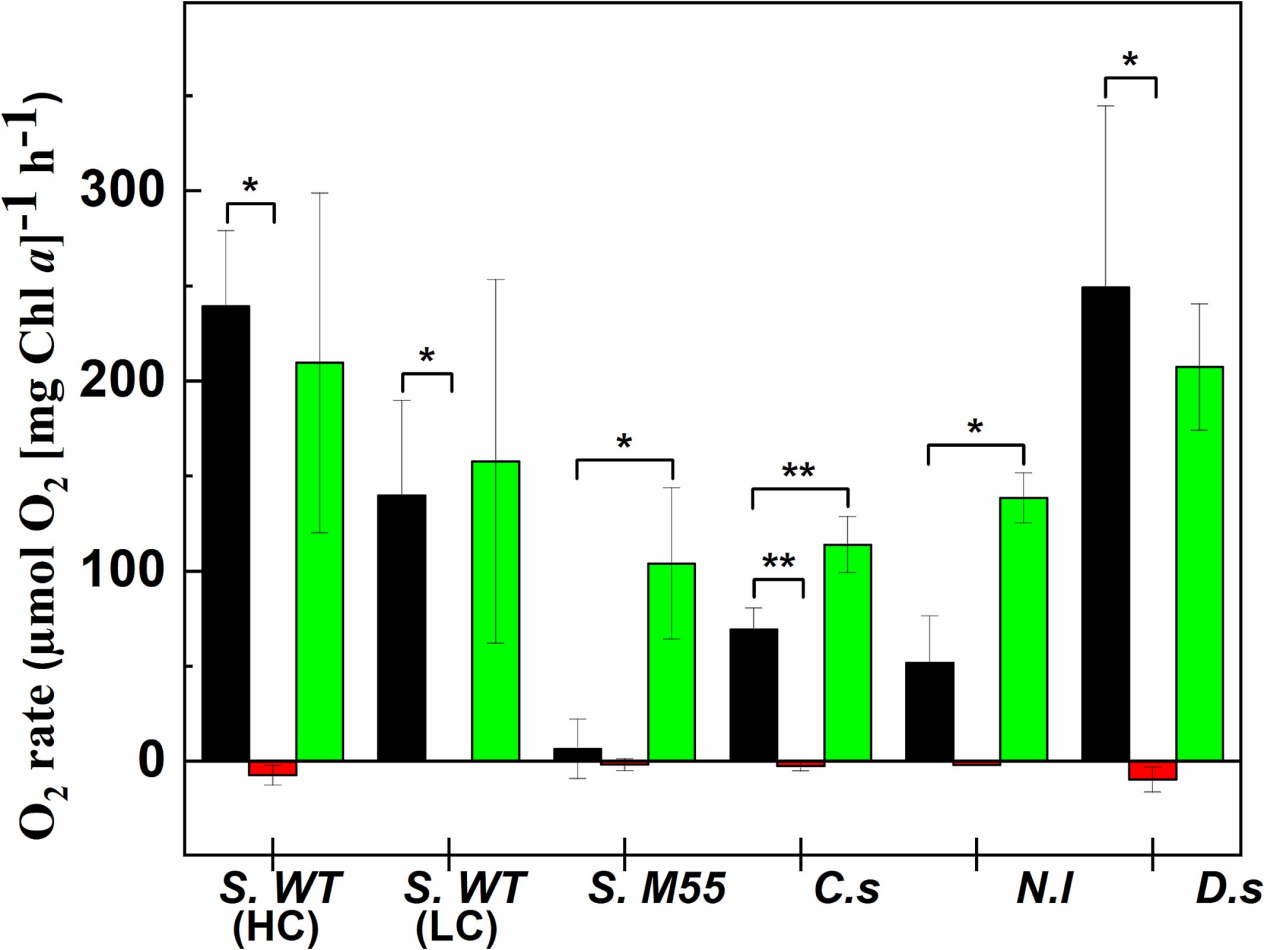
**Rate of O_2_ evolution or uptake measured in Ci-replete (black), -depleted (red) and recovery (green) stages.** Rate of O_2_ was calculated from the linear slope of dissolved O_2_ obtained during Ci limitation (red lines in Fig 2). Values are presented as a mean ± SD (n = 3) indicated by error bars. The asterisks mark significant difference between the indicated states (single asterisks (*): p<0.05, double asterisks (**): p<0.01, triple asterisks (***): p<0.001). Abbreviations: S. WT (HC) (*Synechocystis* sp. PCC6803 WT High CO_2_ grown), S. WT (LC) (*Synechocystis* sp. PCC6803 WT Low (ambient) CO_2_ grown), S. M55 (*Synechocystis* M55 mutant), C.s (*Chlorella sorokiniana*), N.l (*Nannochloropsis limnetica*), D.s (*Dunaliella salina*).

During the course of prolonged illumination significant loss in the O_2_ evolving capacity was observed in all species in Ci-depleted state (Fig 4). Oxygen evolution in Ci-replete state was found to be 239.3±40 μmol O_2_ (mg Chl *a*)^-1^ h^-1^ (all O_2_ rate values are expressed in the same units) in *Synechocystis WT PCC6803* 3% CO_2_ grown samples, 139.7±50 in *Synechocystis WT PCC6803* grown at ambient CO_2_ and 6.55±15.6 in the M55 mutant. Oxygen evolution rate was 69.4±11.25 in *Chlorella sorokiniana*, 51.9±24.6 in *Nannochloropsis limnetica* and 249.4±58.1 in *Dunaliella salina*. Under Ci-limited conditions, O_2_ evolution capacity was essentially lost and negative rates could be observed, indicating that the O_2_ uptake rate exceeded the rate of O_2_ evolution under Ci limitation. The O_2_ evolution capacity was significantly regained in the recovery phase (after addition of NaHCO_3_) in *Synechocystis* grown both at 3% and ambient CO_2_. In the case of the M55 mutant, O_2_ evolution after NaHCO_3_ addition was significantly higher, 104.07±39.7 (p*<0*.*05*) as compared to the initial ‘Ci-replete’ stage, indicating that the M55 mutant was heavily Ci-limited during growth and at the beginning of the experiment. In microalgae, O_2_ evolution recovered to 113.9±14.7 (p*<0*.*01*) in *C*. *sorokiniana*, to 138.56±13.18(p*<0*.*05*) in *N*. *limnetica*, and to 207.4±33.3 in *D*. *salina*.

### NADPH fluorescence kinetics under Ci limitation

NADPH fluorescence kinetics is a versatile probe to investigate several photosynthetic processes downstream of PSI, the operation of ferredoxin NADP^+^ oxidoreductase (FNR), activation and limitations of the Calvin-Benson cycle. This method has been proven especially useful in the model cyanobacterium *Synechocystis* [19, 37, 38, 50] and the green algal model organisms *Chlorella vulgaris* [38] and *Chlamydomonas reinhardtii* [51, 52], but rarely applied for diagnostic purposes in other microalgae that have high relevance in applied research. As employed here, the integrated cuvette-based photosynthesis analysis system allows recording NADPH kinetics without disturbing the physiological state of the sample or changing the geometry of the setup, therefore screening of NADPH fluorescence-based traits can be performed at customarily selected time points of the experiment. Initial fluorescence was measured in the dark for 30 s to obtain the steady state level of the NADP pool. An initial rise in NADPH fluorescence at the onset of actinic illumination represents light-driven NADP reduction via linear electron flow, which is followed by a pronounced dip phase, before a second rise phase sets in (the complex phenomenon of the initial NADPH fluorescence kinetics is analyzed and described in [19, 37–39]). NADPH fluorescence exhibits a local maximum at around about 20 s (Nm), followed by a fluorescence decrease due to NADPH consumption by activation of the CBB cycle for CO_2_ fixation and then attains a plateau phase during which NADPH production and consumption are in equilibrium (Nt). Under Ci-replete condition, NADPH fluorescence declines from the Nm to Nt phase within about 50 s, indicating the high uptake capacity of these NADPH-utilizing pathways. After turning off the actinic light, a sharp decline in NADPH fluorescence could be observed, as LEF ceases to drive electrons for reducing NADP^+^, but the NADPH consumption pathways remain active due to the uptake of NADPH via the CBB cycle, resulting in an undershoot of the NADPH signal. The ‘undershoot’ signal plateaued within about 15–20 s, in agreement with the time course of cessation of CO_2_ assimilation in the darkness after illumination (e.g. [53]). Finally, NADPH fluorescence recovers to the initial dark-adapted state due to the filling up of the NADPH pool e.g. via the reductive pentose phosphate pathway [37].

**Fig 5:**
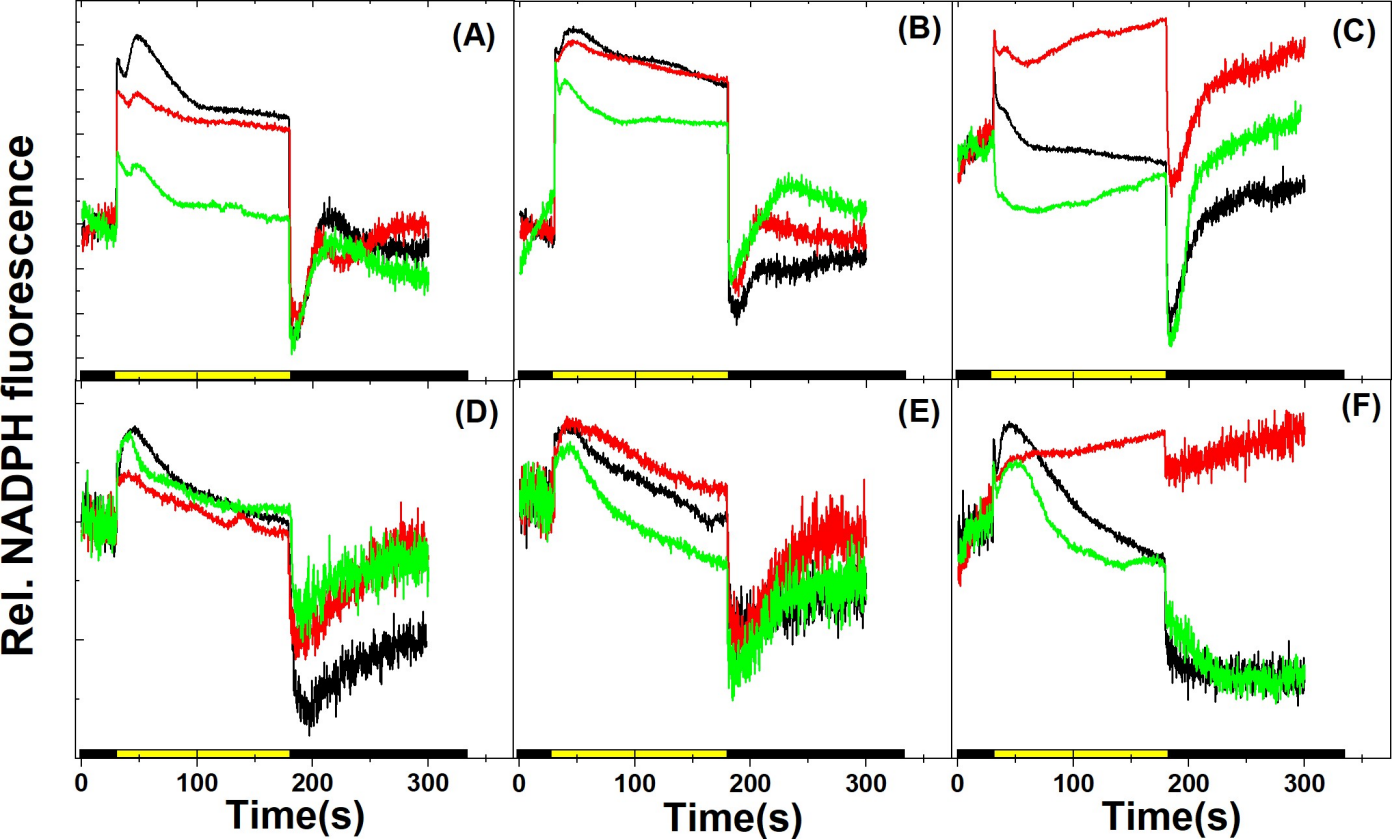
**NADPH fluorescence kinetics traces in Ci-replete (black), -depleted (red) and recovery (green) states during the course of Ci limitation, for all species and conditions.** Cells were dark-adapted for 3 min before each measurement. Data acquisition was started in the dark, followed by illumination with actinic red light at ~56 μmol photons m^-2^ s^-1^, and was finished in darkness, as indicated by the black, yellow, and black bars on the X axis, respectively. The first 30 s of traces were measured in the dark, actinic light was turned on at 30 s and turned off at 180 s, and the light-to-dark changes were measured up to 300 s. (A) *Synechocystis* sp. PCC6803 WT grown at 3% CO_2_, (B) *Synechocystis* sp. PCC6803 WT grown at ambient CO_2_, (C) *Synechocystis M55* mutant, (D) *Chlorella sorokiniana*, (E) *Nannochloropsis limnetica*, (F) *Dunaliella salina*. Each trace represents the average of three replicates.

In *Synechocystis* grown at 3% CO_2_, a marked change could be observed during the transition from Ci-replete to -depleted stage; the decline from Nm to Nt was less expressed, indicating the decreased NADPH uptake capacity by the CBB cycle (Fig 5A). This was partially recovered after adding 10 mM NaHCO_3_, and the overall NADPH fluorescence intensity was also observed to decrease, probably because of the elevated rate of NADPH consumption over NADPH production upon repletion of the cells with Ci [19]. *Synechocystis* cells grown at ambient CO_2_ (Fig 5B) showed an elevated NADPH fluorescence in the initial Ci-replete state (resuspended in BG11 medium), the first rapid dip in fluorescence was diminished, and the Nm-Nt fluorescence difference decreased, indicating that the cells entered the Ci-limited stage. Under prolonged Ci limitation, the first dip in NADPH fluorescence was absent. Addition of NaHCO_3_ restored the Ci-replete ‘phenotype’, as the fast fluorescence dip reappeared and the Nm-Nt difference became more pronounced (and the overall NADPH fluorescence decreased), indicating a regained CBB cycle activity based on NADPH fluorescence signature (Fig 5B). It has to be noted that the initial rapid changes in NADPH fluorescence kinetics (in the first 50 s timescale after the onset of AL) cannot be assigned to the activity of the CBB cycle, because the activation of the CBB cycle is a slower reaction; Rubisco activation and CO_2_ assimilation rates attain their maxima within a few minutes [54, 55]. Moreover, application of the CBB cycle inhibitor glycolaldehyde (GA) did not affect NADPH fluorescence kinetics significantly in the short (<min) timescale but inhibited the NADPH fluorescence decrease after the Nm phase, indicating that NADPH uptake by the CBB cycle was impaired (S9 Fig).

In the case of the M55 mutant (Fig 5C), AL caused only a minor transient increase in NADPH fluorescence, but the NADPH fluorescence level rapidly decreased below the initial dark-adapted level, and a rapid decrease after illumination also occurred. In Ci-limited stage, the light-induced NADPH fluorescence increase was present, however the dip during the illumination period was diminished, consequently Nt was at a much higher level than in the Ci-replete control. After replenishing M55 cells with NaHCO_3_, the initial Ci-replete ‘phenotype’ was even more expressed, i.e. the complete lack of the light-induced transient increase was accompanied with a very pronounced decrease below the initial level during AL and the undershoot of the NADPH fluorescence signal after illumination. The absence of NADPH uptake by the CBB cycle during actinic illumination in Ci-depleted phase is in agreement with previous studies in the M55 mutant; it was observed that when the CBB cycle was chemically blocked, light-induced NADPH formation was not significantly influenced, but NADPH uptake during light in 1–2 min timescale was retarded [37]. However, the current study also reveals that in the Ci-limited phase in the M55 mutant the light-induced increase in NADPH fluorescence was more expressed than in the Ci-replete state. This is possibly due to the fact that in M55 the NADPH level in the dark was lower in Ci-depleted than in Ci-replete state (and so, the ‘phenotype’ of Ci-limited M55 cells resembled the ‘phenotype’ of WT cells in Ci-depleted state).

The NADPH fluorescence phenomenology exhibited similar patterns in eukaryotic microalgae as well; the characteristic initial rise, a transient dip, a secondary rise to a peak (Nm) followed by a decrease upon CBB activation could be observed, in agreement with previous studies on NADPH fluorescence kinetics in *C*. *vulgaris* [38]; however, minor differences in different species could also be identified. Light-induced NADPH fluorescence changes were the most sensitive to Ci limitation in *D*. *salina* (Fig 5F). The fluorescence signal nearly flattened upon Ci depletion with significant loss in both light-induced NADPH fluorescence increase and Nm-Nt difference, and the post-illumination fluorescence decrease was diminished as well. These changes were nearly completely reversible upon Ci repletion. In the species *C*. *sorokiniana* and *N*. *limnetica* (Fig 5D and 5E, respectively) light-induced NADPH fluorescence kinetics and its changes during transition from Ci-replete to -depleted state (and recovery after NaHCO_3_ addition) exhibited similar properties (i.e. the flattening of NADPH fluorescence from Nm to Nt under Ci depletion, which was partially reversible after addition of NaHCO_3_). However, these changes were less characteristic as compared to *D*. *salina* and *Synechocystis*, even though the onset of Ci limitation based on PSII quantum yield and cessation of oxygen evolution (see above) was evident. This indicates that alternative NADPH oxidation mechanisms might be operational even when NADPH oxidation by the CBB cycle under Ci limitation is hampered (e.g. [56]), and these multiple mechanisms might contribute to NADPH uptake kinetics to different extents in the different species. Therefore, although the NADPH fluorescence phenomenology is a sensitive marker of rapid changes in the Ci status of cells, further studies are required to fully elucidate its applicability under various physiological conditions in green and eustigmatophycean algae.

### Chlorophyll fluorescence induction-recovery kinetics under Ci limitation

**Fig 6:**
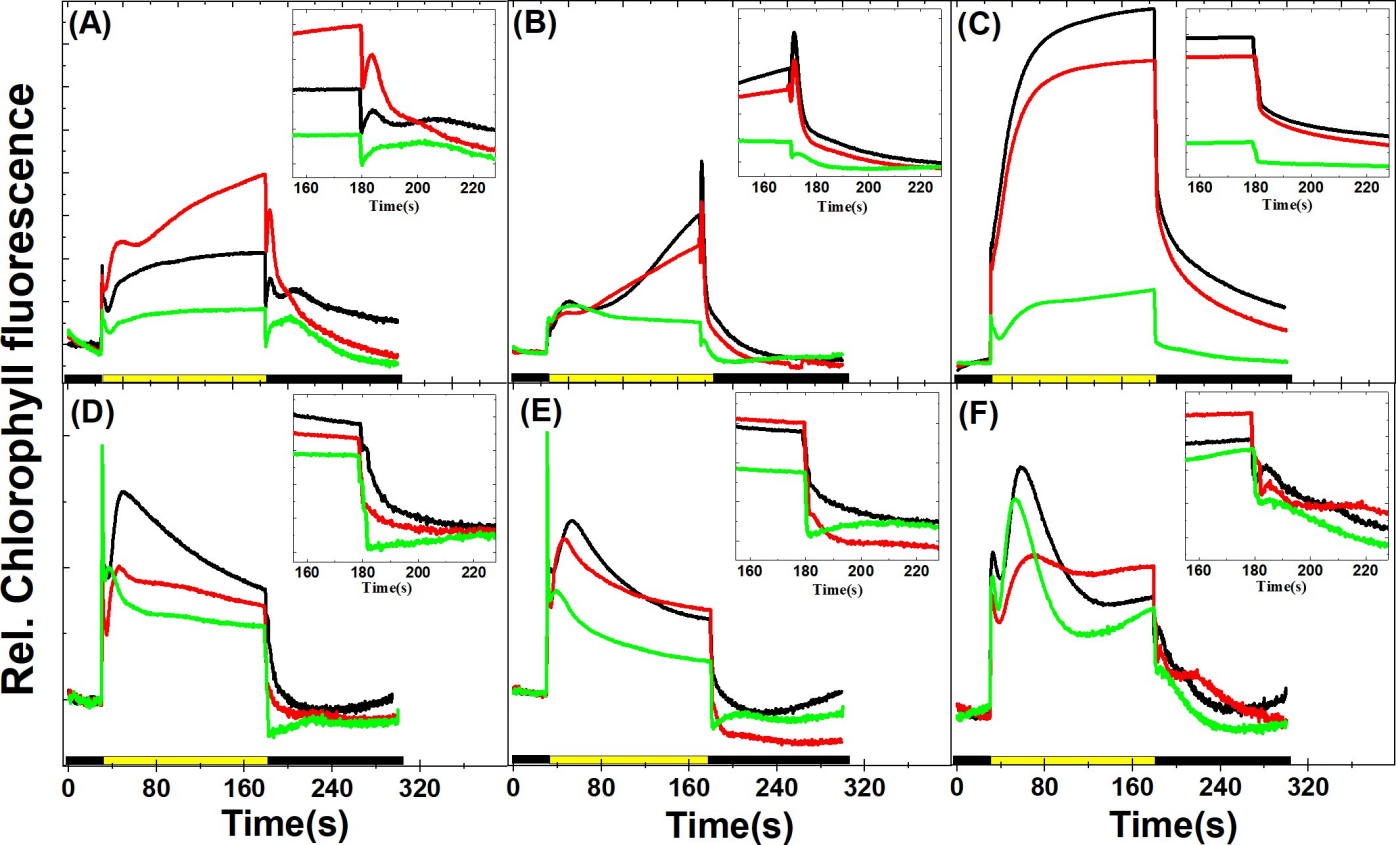
**Chlorophyll fluorescence induction kinetics traces in Ci-replete (black), -depleted (red) and recovery (green) states during the course of Ci limitation.** The first 30 s of traces were measured in the dark, actinic light (~56 μmol photons m^-2^ s^-1^) was turned on at 30 s and turned off at 180 s, and subsequently post-illumination kinetics was measured up to 300 s. (A) *Synechocystis* sp. PCC6803 WT grown at 3% CO_2_, (B) *Synechocystis* sp. PCC6803 WT grown at ambient CO_2_, (C) *Synechocystis M55* mutant, (D) *Chlorella sorokiniana*, (E) *Nannochloropsis limnetica*, (F) *Dunaliella salina*. The insets in each panel show the post-illumination chlorophyll fluorescence rise during the light to dark transition period. Each trace represents the average of three replicates.

Chlorophyll fluorescence kinetics were recorded in parallel with NADPH fluorescence kinetics, therefore this combined approach allowed a close monitoring of changes in photosynthetic electron transport under Ci limitation. After switching on the actinic light, an increase in chlorophyll fluorescence was observed, the kinetics of which was complex and the underlying phenomena are not fully resolved here. In general, it is related to progressive reduction and reoxidation of quinone electron acceptors (mainly Q_A_ and PQ), which reflects a quasi-steady state balance of rates corresponding to the actinic excitation rate generating Q_A_^-^ (Q_A_ reduction rate) and the rate of forward electron transfer of electron into the PQ pool via the PSII Q_B_ site (Q_A_^−^oxidation rate) [57, 58]. Upon prolonged illumination (30 s– 150 s) the chlorophyll fluorescence declines, as PQ becomes oxidized due to the activation of photochemical quenching and the CBB cycle (this phase also corresponds to the initiation of NADPH uptake by the CBB cycle (see Fig 5). Upon switching off the actinic light at 180 s, chlorophyll fluorescence displays fluctuations with multiple peaks on different timescales, which are related to the redox changes in the PQ pool, mediated by the electron transport carriers from the cytosol to the chloroplast stroma [19, 59, 60]. The fast phase of post-illumination rise was observed within 1–10 s after actinic light was switched off in *Synechocystis* PCC 6803 WT: this post-illumination rise can be attributed to the operation of the cyclic electron flow [19]. In *Synechocystis* PCC 6803 WT (grown at 3% CO_2_) the post-illumination rise was observed within 3–5 s in both in Ci-replete and -depleted state, and it was diminished in the Ci recovery phase (Fig 6A). *Synechocystis* cells grown at ambient CO_2_ expressed faster post-illumination rise kinetics within 1–3 s (in agreement with [60]), the amplitude of which, however, did not change during the prolonged Ci limitation phase (Fig 6B). Post-illumination fluorescence rise was completely missing in the mutant M55, irrespectively of Ci content (Fig 6C), in agreement with previous studies (e.g. [61]). A slower post-illumination fluorescence rise signal in the timescale of 20–50 s was also observed earlier in *Synechocystis* and was denoted as F_R_, which could be assigned to enhanced reductant flow to the PQ pool because of the accumulation of reduced carbon skeletons from the operation of the CBB cycle [59]. *Synechocystis* grown at 3% CO_2_ displayed this slow rise in the initial Ci-replete stage, but it was particularly expressed after addition of NaHCO_3_ after the Ci-depleted phase (inset of Fig 6A, in agreement with [59]). In *Synechocystis* grown at ambient CO_2_ this slow rise was less expressed, as the post-illumination rise was dominated by the faster component, likely attributable to elevated CEF. After addition of NaHCO_3_, the fast post-illumination component disappeared and, interestingly, the slow F_R_ peak could not be observed either. However, a smaller and faster post-illumination component remained (Fig 6B inset), indicating altered reductant flow under ambient CO_2_ cultivation and possibly also due to a higher reduction state of the PQ pool (see below). Based on the above findings, Chl fluorescence characteristics at different stages of actinic illumination and after cessation of illumination could be a simple indicator of Ci status and possibly also the indicator of the PQ pool’s redox state mediated by the carbohydrate biosynthesis pathways in cyanobacteria.

The Chl fluorescence induction kinetics was more complex in microalgae, and the results presented in the current study do not allow a detailed interpretation of the underlying mechanisms. Nevertheless, in general it can be concluded that the fast post-illumination rise signal, indicative of elevated CEF capacity under Ci limitation, could not be observed in *C*. *sorokiniana* and *N*. *limnetica* (Fig 6D and 6E, respectively), possibly because alternative mechanisms (pseudo-cyclic electron transport mechanisms, Mehler reaction, plastidial terminal oxidase) compete with CEF and therefore CEF-mediated PQ reduction is alleviated. *D*. *salina* (Fig 6F) exhibited a small but apparent post-illumination rise, which was more pronounced in Ci-depleted phase and disappeared after NaHCO_3_ treatment. The appearance of the slow component of the fluorescence rise (20–40 s) after addition of NaHCO_3_ was apparent in *C*. *sorokiniana* and *N*. *limnetica* (inset of Fig 6D and 6E, respectively). The multi-phasic fluorescence oscillation pattern appeared to behave differently in *D*. *salina*; in this species a slow component could also be observed in Ci-limited phase, whereas in the initial and recovery phases fluorescence decreased monotonically without exhibiting any post-illumination peaks (Fig 6F inset). Therefore, the multiple components of fluorescence oscillations could be employed for obtaining information about the rapid regulation of electron transport (such as CEF), as well as the redox changes associated with carbohydrate metabolism in eukaryotic algae.

### Analysis of forward electron transfer kinetics under Ci limitation

**Fig 7:**
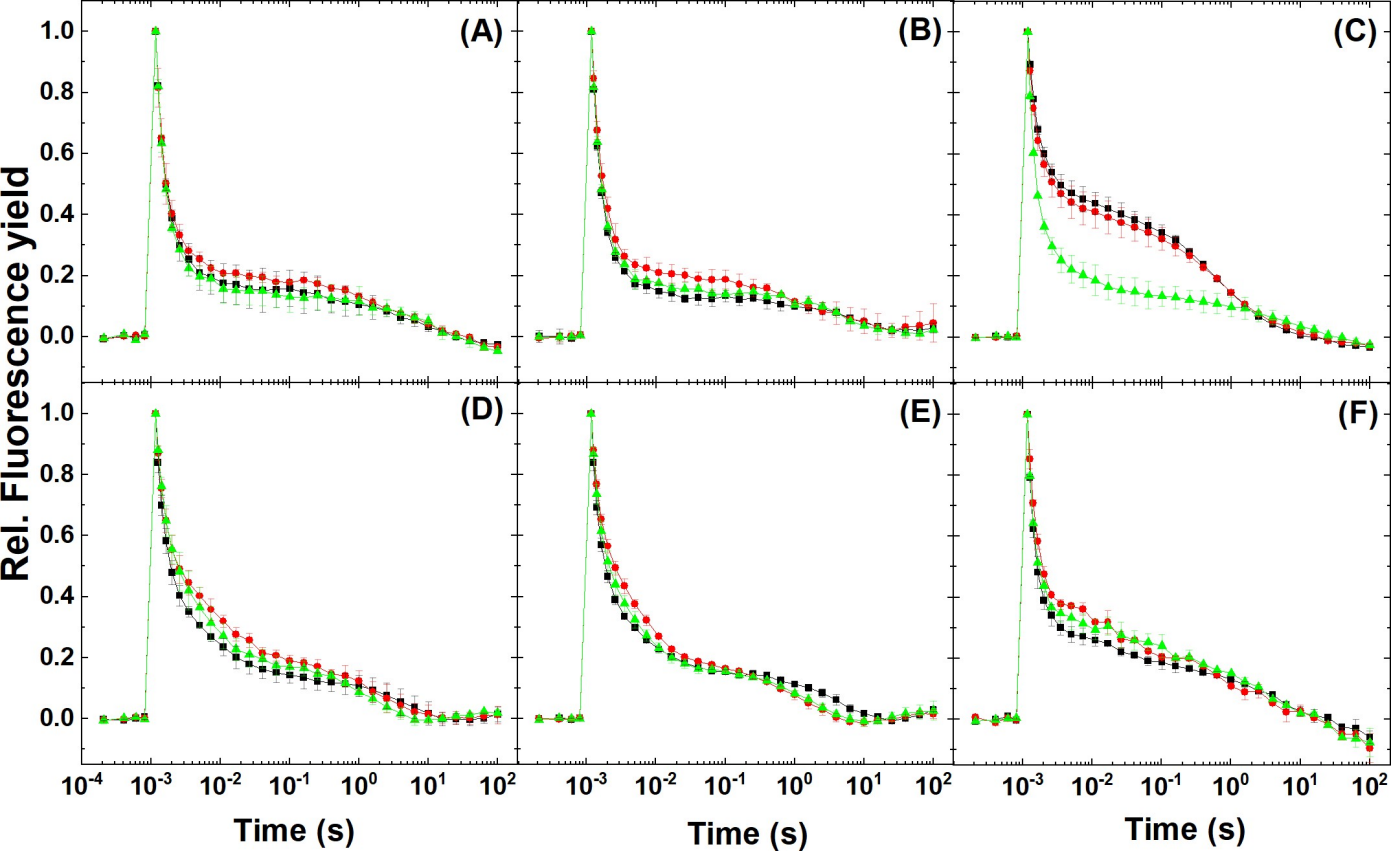
**Flash-induced chlorophyll fluorescence decay curve traces at Ci-replete (black), -depleted (red) and recovery (green) stages undergoing Ci limitation.** (A) *Synechocystis* sp. PCC6803 WT grown at 3% CO_2_, (B) *Synechocystis* sp. PCC6803 WT grown at ambient CO_2_, (C) *Synechocystis M55* mutant, (D) *Chlorella sorokiniana*, (E) *Nannochloropsis limnetica*, (F) *Dunaliella salina*. Traces represent the average of 3 replicates.

Flash-induced chlorophyll fluorescence decay curves give information about the re-oxidation kinetics of Q_A_^-^ via forward electron transfer to Q_B_ and the PQ pool, or recombination with the donor side of PSII. The relaxation is dominated by a fast phase (~300–500 μs) that reflects electron transfer from Q_A_^−^ to Q_B_ (Q_B_^−^). The middle phase (~5–15 ms) arises from reoxidation of Q_A_^−^ by PQ that binds to the Q_B_ site after flash, whereas the slow phase (~10–20 s) arises from the S_2_Q_A_Q_B_^−^ charge recombination [15]. Flash-induced fluorescence decay profiles showed a slightly higher fluorescence level in Ci-depleted stage in *Synechocystis* PCC6803 WT (grown with and without CO_2_) on the 5–500 ms timescale (Fig 7A and 7B). In the M55 mutant (Fig 7C) the fluorescence decay was slow and the fluorescence level remained high in the 2 ms-1 s timescale in the initial Ci-replete state and the progression to Ci-limited stage did not change this pattern, indicating that the electron transport capacity was limited, in agreement with the low effective quantum yield of PSII (Fig 3) and O_2_ evolution capacity (Fig 4) under this condition. However, a fast decay is regained after addition of NaHCO_3_, in agreement with the restoration of linear electron flow and O_2_ evolving capacity (c.f. Figs 3 and 4, respectively). *Chlorella sorokiniana* and *Dunaliella salina* in the 2 ms-1 s region (Fig 7D and 7F, respectively) and *Nannochloropsis limnetica* in the 2–20 ms region (Fig 7E) showed similar patterns after Ci limitation, which indicated a limited capacity of reoxidation of Q_A_^-^ by the PQ molecules bound to the Q_B_ site after the flash [15] under Ci-limited condition. These changes were largely reversible after addition of NaHCO_3_, in agreement with previous studies that showed the restoration of electron flow between Q_A_^-^ to Q_B_ after addition of 10 mM NaHCO_3_ to bicarbonate-depleted cells [62, 63]. Taken together, flash fluorescence decay signals indicated slightly decreased capacity of Q_A_^-^ reoxidation by the PQ molecules under Ci-limited condition in all species and conditions, although these changes appeared to be minor under the conditions investigated here.

**Fig 8:**
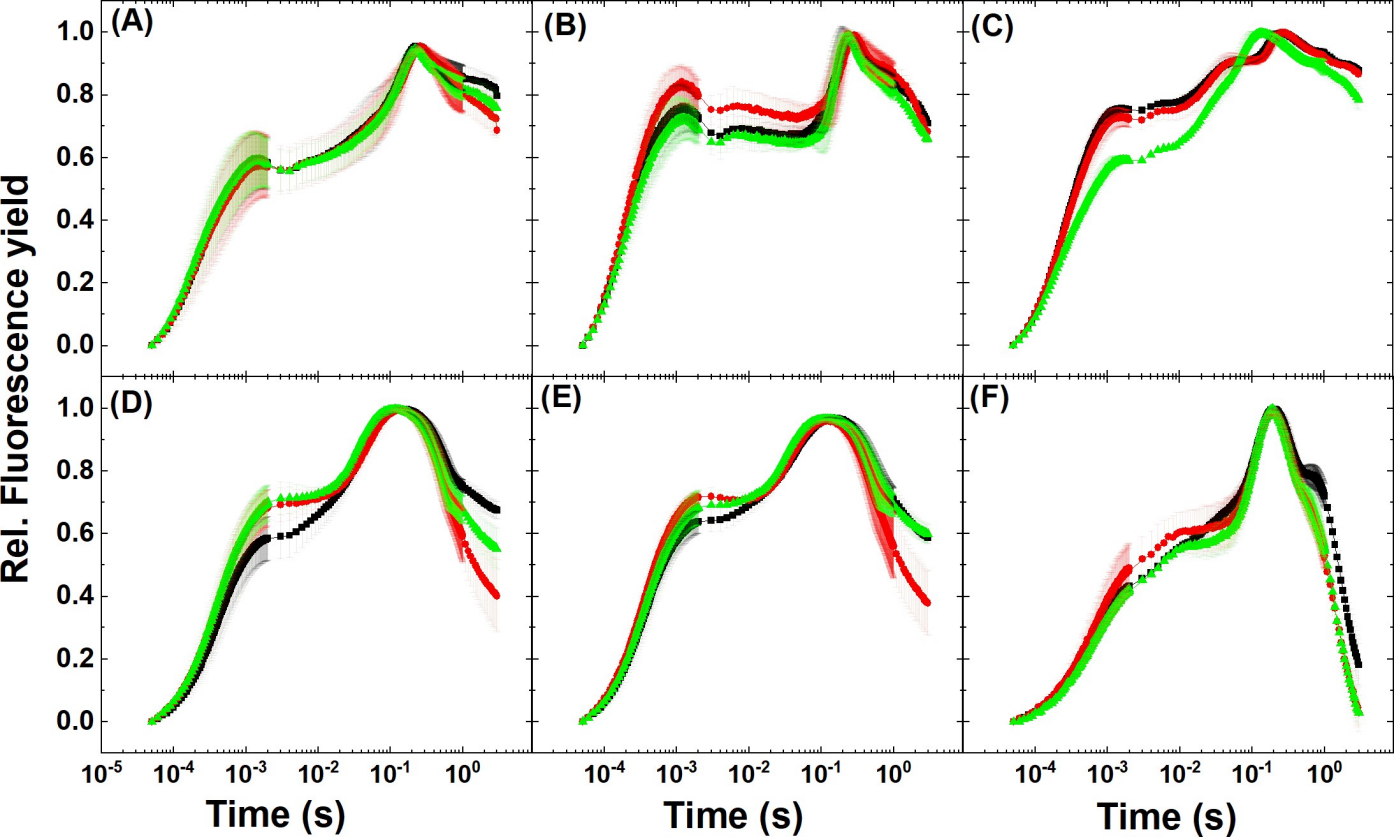
**OJIP transients at Ci-replete (black), -depleted (red) and recovery (green) stage undergoing Ci limitation.** Cells were dark-adapted for 3 min before each measurement. (A) *Synechocystis* sp. PCC6803 WT grown at 3% CO_2_, (B) *Synechocystis* sp. PCC6803 WT grown at ambient CO_2_, (C) *Synechocystis M55* mutant, (D) *Chlorella sorokiniana*, (E) *Nannochloropsis limnetica*, (F) *Dunaliella salina*. Traces represent the average of 3 replicates.

The polyphasic rise of chlorophyll fluorescence, or commonly referred to as OJIP curves, is a frequently used method to investigate fast dynamic changes in various components of the photosynthetic electron transport chain and PSII efficiency from dark to light transition (e.g. [64, 65]). Transient chlorophyll *a* fluorescence rise is induced during a dark-to-strong light transition, where O is equivalent to F_0_, P is for peak, equivalent to F_m_ (when measured at saturating light) and J and I (or F_J_ and F_I_) are inflection points typically at 2 and 30 ms, respectively, between O and P. The O-J fluorescence rise is the photochemical phase reflecting the closure of open reaction centers, where the PSII electron acceptors Q_A_ and Q_B_ become fully reduced, and the reduction of Q_A_ and the reoxidation of Q_A_^-^ by PQ molecules are in equilibrium at the J step (2–3 ms). The J-I phase is a dip due to the partial reduction of intersystem electron carriers Q_B_, PQ, Cyt b_6_/f (mainly the PQ pool) and the electron transfer towards the PSI, and the I-P phase is determined by electron carriers PQH_2_, PC^+^, P700^+^ and PSI electron acceptors (such as FNR activity) [66, 67]). In ambient CO_2_-grown *Synechocystis* (Fig 8B), the J phase was relatively high as compared to *Synechocystis* grown at 3% CO_2_ (Fig 8A), indicating a slower Q_A_^-^ reoxidation by PQ molecules. The onset of Ci limitation from the initial Ci-replete state caused an elevated J step, which was reversible after NaHCO_3_ addition. The increase in J step under Ci limitation was not significant in 3% CO_2_ grown *Synechocystis*. The M55 mutant (Fig 8C) already exhibited a high J step in the Ci-replete state, which did not change in Ci-limited state; however, it significantly dropped after NaHCO_3_ addition as a result of the restoration of active electron transport (c.f. Figs 3, 4 and 7 and also S3 Fig). The increase in J step under Ci limitation was also observed in eukaryotic microalgae (Fig 8D–8F), which partially or fully recovered upon NaHCO_3_ addition, although the changes in the O-J-I-P phases appeared to be significant only in the *Synechocystis* M55 mutant after NaHCO_3_ addition (the original OJIP transients and transients normalized to the F_0_ level are shown in Supplementary Material S10 and S11 Figs). These results indicate that the OJIP transients are informative to reveal species- and condition-specific patterns, although the induced CO_2_ limitation exerted only minor effects on Q_A_^-^ reoxidation, except in the M55 mutant of *Synechocystis* (in agreement with the flash-induced fluorescence decay patterns, Fig 7).

## Conclusions

The cuvette system presented in the current study enabled simultaneous real-time monitoring of several parameters that are indicative of the physiological status of the photosynthetic apparatus of cyanobacteria and microalgae. Photosynthetic characterization of the frequently applied model cyanobacterium *Synechocystis* sp. PCC6803 under transient CO_2_ limitation showed that the integrated cuvette system is applicable to decipher distinct traits of this model species grown under different CO_2_ regimes (also in agreement with some of the findings by [13, 19]). Moreover, the ndhB-deficient M55 mutant, which lacks all NDH-1 complexes (and is therefore unable to perform CEF and CCM) exhibited very clear phenotypic differences in Ci-replete vs. -depleted state. Although the M55 mutant has been thoroughly characterized in the past (e.g. [37, 68–70] reviewed in [71, 72]), applying this mutant in our studies was an important validation of the suitability of real-time monitoring of several photosynthetic parameters (changes in O_2_ and Y(II) dynamics), along with alterations of NADPH synthesis/uptake kinetics, the redox state of the PQ pool and forward electron transfer kinetics in different Ci conditions. Therefore, this system is potentially applicable to the detection of subtle real-time changes in other *Synechocystis* mutants. Our results with *Synechocystis* allowed to extend physiological monitoring to other species of eukaryotic algae, by measuring not only the absolute values of PSII quantum yield and/or oxygen evolution rates at arbitrarily selected time points, but also the dynamic changes of photosynthetic parameters in algal suspensions subjected to various inorganic carbon regimes (e.g. onset, early and prolonged Ci limitation and kinetics of recovery from these conditions upon Ci repletion). In addition to the comprehensive monitoring of photosynthetic parameters and recording the extracellular pH in the medium (using a pH microsensor), the integrated cuvette system presented here can be extended with highly sensitive detection of CO_2_ (using membrane inlet mass spectrometry) and dynamic modelling of carbon chemistry. In this way the exchange of inorganic carbon species and the details of the inorganic carbon cycling (e.g. [73]) could also be revealed in future studies. These integrative approaches would therefore facilitate screening for stress- or condition-specific traits in industrially important microalgae and cyanobacteria.

## Supporting information

S1 Table Original absorbance readings of acetone:DMSO pigment extracts and the calculated Chl *a* and Chl *b* content, total Chl (*a+b*) content and the Chl *a/b* ratio. Eq 1 was used for calculating Chl *a* and Eq 2 was used for calculating Chl *b*.
Chl *a* (μg mL^-1^) = 11.75*A_663_−2.35*A_645_                   (1)
Chl *b* (μg mL^-1^) = 18.61*A_645_−3.96*A_663_                   (2)
^#^In *N*. *limnetica* the calculated Chl *b* content and Chl a/b ratio cannot be accurately interpreted due to the lack of Chl *b*.
(DOCX)

S1 Fig Representative graph of physiological response of *Synechocystis* sp. PCC 6803 WT (3% CO_2_ grown) during the course of Ci limitation. Simultaneous measurements of (A) Oxygen evolution and (B) Effective quantum yield of PSII (Y(II)) and (C) pH were performed. Y(II) and pH was measured every 5 min. The cells were kept in the dark for 30 min (grey shade region) then illuminated with actinic white light at 56 μmol photons m^-2^ s^-1^. The dots in panel A represent the defined measuring time points of chlorophyll and NADPH fluorescence kinetics at: T0 (control) after 3 min in the dark; T1—initial Ci-replete phase (30 min) in the light; T2—early phase of Ci depletion (135 min); T3—prolonged Ci-depleted phase (240 min); T4—Ci recovery phase (265 min) in the light (arrow indicates the addition of 10 mM NaHCO_3_).
(DOCX)

S2 Fig Representative graph of physiological response of *Synechocystis* sp. PCC 6803 WT (Ambient CO_2_ grown) during the course of Ci limitation. (A) Simultaneous measurements of A) Oxygen evolution and (B) Effective quantum yield of PSII (Y(II)) and (C) pH were performed. Y(II) and pH was measured every 5 min. The cells were kept in the dark for 25 min (grey shade region) then illuminated with actinic white light at 56 μmol photons m^-2^ s^-1^. The dots in panel A represent the defined measuring time points of chlorophyll and NADPH fluorescence kinetics at: T0 (control) after 3 min in the dark; T1—initial Ci-replete phase (50 min in the light); T2—early phase of Ci depletion (125 min in the light); T3—prolonged Ci-depleted phase (230 min); T4—Ci recovery phase (270 min) in the light (arrow indicates the addition of 10 mM NaHCO_3_).
(DOCX)

S3 Fig Representative graph of physiological response of *Synechocystis* M55 mutant during the course of Ci limitation. Simultaneous measurements of (A) Oxygen evolution and (B) Effective quantum yield of PSII (Y(II)) and (C) pH were performed. Y(II) and pH was measured every 5 min. The cells were kept in the dark for 20 min (grey shade region) then illuminated with actinic white light at 56 μmol photons m^-2^ s^-1^. The dots in panel A represent the defined measuring time points of chlorophyll and NADPH fluorescence kinetics at: T0 (control) after 3 min in the dark; T1—initial Ci-replete phase (55 min) in the light; T2—prolonged Ci-depleted phase (125 min in the light); T3—Ci recovery phase (175 min) in the light (arrow indicates the addition of 10 mM NaHCO_3_).
(DOCX)

S4 Fig Representative graph of physiological response of *Chlorella sorokiniana* the course of Ci limitation. Simultaneous measurements of (A) Oxygen evolution and (B) Effective quantum yield of PSII (Y(II)) and (C) pH were performed. Y(II) and pH was measured every 5 min. The cells were kept in the dark for 20 min (grey shade region) then illuminated with actinic white light at 56 μmol photons m^-2^ s^-1^. The dots in panel A represent the defined measuring time points of chlorophyll and NADPH fluorescence kinetics at: T0 (control) after 3 min in the dark; T1—initial Ci-replete phase (80 min) in the light; T2—early phase of Ci depletion (170 min); T3—prolonged Ci-depleted phase (300 min); T4—Ci recovery phase (335 min) in the light (arrow indicates the addition of 10 mM NaHCO_3_).
(DOCX)

S5 Fig Representative graph of physiological response of *Nannochloropsis limnetica* during the course of Ci limitation. Simultaneous measurements of (A) Oxygen evolution and (B) Effective quantum yield of PSII (Y(II)) and (C) pH were performed. Y(II) and pH was measured every 5 min. The cells were kept in the dark for 20 min (grey shade region) then illuminated with actinic white light at 56 μmol photons m^-2^ s^-1^. The dots in panel A represent the defined measuring time points of chlorophyll and NADPH fluorescence kinetics at: T0 (control) after 3 min in the dark; T1—initial Ci-replete phase (125 min) in the light; T2—early phase of Ci depletion (240 min); T3—prolonged Ci-depleted phase (370 min); T4—Ci recovery phase (420 min) in the light (arrow indicates the addition of 10 mM NaHCO_3_).
(DOCX)

S6 Fig Representative graph of physiological response of *Dunaliella salina* during the course of Ci limitation. Simultaneous measurements of (A) Oxygen evolution and (B) Effective quantum yield of PSII (Y(II)) and (C) pH were performed. Y(II) and pH was measured every 5 min. The cells were kept in the dark for 40 min (grey shade region) then illuminated with actinic white light at 56 μmol photons m^-2^ s^-1^. The dots in panel A represent the defined measuring time points of chlorophyll and NADPH fluorescence kinetics at: T0 (control) after 3 min in the dark; T1—initial Ci-replete phase (85 min) in the light; T2—early phase of Ci depletion (180 min); T3—prolonged Ci-depleted phase (280 min); T4—Ci recovery phase (335 min) in the light (arrow indicates the addition of 10 mM NaHCO_3_).
(DOCX)

S7 Fig Oxygen evolution rate and electron transport rate (ETR) during the course of Ci limitation. Representative traces of the first derivatives of oxygen evolution (O_2_, black) and ETR (red). The blue arrow represents the time of NaHCO_3_ addition. (A) *Synechocystis* sp. PCC 6803 WT grown at 3% CO_2_, (B) *Synechocystis* sp. PCC 6803 WT grown at ambient CO_2_, (C) *Synechocystis* M55 mutant, (D) *Chlorella sorokiniana*, (E) *Nannochloropsis limnetica*, (F) *Dunaliella salina*
(DOCX)

S8 Fig Representative chlorophyll fluorescence traces during the course of Ci limitation. The blue arrow represents the time of NaHCO_3_ addition. (A) *Synechocystis* sp. PCC 6803 WT grown at 3% CO_2_, (B) *Synechocystis* sp. PCC 6803 WT grown at ambient CO_2_, (C) *Synechocystis* M55 mutant, (D) *Chlorella sorokiniana*, (E) *Nannochloropsis limnetica*, (F) *Dunaliella salina*
(DOCX)

S9 Fig **Representative NADPH fluorescence traces of WT *Synechocystis* sp. PCC 6803 WT in the absence (black line) or presence (red line) of 10 mM GA.** Cells were grown in the presence of 3% CO_2_ (as described in Materials and Methods) and dark adapted for 5 min before the measurements.
(DOCX)

S10 Fig **Representative original OJIP traces (not normalized) at Ci-replete (black), -depleted (red) and recovery (green) stage undergoing Ci limitation.** Cells were dark adapted 3min before every measurement. (A) *Synechocystis* sp. PCC 6803 WT grown at 3% CO_2_, (B) *Synechocystis* sp. PCC 6803 WT grown at ambient CO_2_, (C) *Synechocystis* M55 mutant, (D) *Chlorella sorokiniana*, (E) *Nannochloropsis limnetica*, (F) *Dunaliella salina*.
(DOCX)

S11 Fig **Representative OJIP traces that were normalized to F**_**0**_ **level (at 50 μs timepoint) at Ci-replete (black), -depleted (red) and recovery (green) stage undergoing Ci limitation.** Cells were dark adapted 3min before every measurement. (A) *Synechocystis* sp. PCC 6803 WT grown at 3% CO_2_, (B) *Synechocystis* sp. PCC 6803 WT grown at ambient CO_2_, (C) *Synechocystis* M55 mutant, (D) *Chlorella sorokiniana*, (E) *Nannochloropsis limnetica*, (F) *Dunaliella salina*.
(DOCX)
