# Supplementary material for: A multi-parametric screening platform for photosynthetic trait characterization of microalgae and cyanobacteria under inorganic carbon limitation
Source: PLoS One. 2020 Jul 23;15(7):e0236188. doi: 10.1371/journal.pone.0236188 (PMC7377499; doi:10.1371/journal.pone.0236188)
Supplement: S1 Table — Eq 1 was used for calculating Chl a and Eq 2 was used for calculating Chl b. Chl a (μg mL-1) = 11.75*A663−2.35*A645 (1) Chl b (μg mL-1) = 18.61*A645−3.96*A663 (2) #In N. limnetica the calculated Chl b content and Chl a/b ratio cannot be accurately interpreted due to the lack of Chl b. (DOCX) [file pone.0236188.s001.docx]

| Sample | A_663_ | A_645_ | Chl *a*  (µg mL^-1^) | Chl *b*  (µg mL^-1^) | Chl (*a+b*) (µg mL^-1^) | Chl *a*/*b* |
| --- | --- | --- | --- | --- | --- | --- |
| *Chlorella sorokiniana* | 0.259 ± 0.01 | 0.101 ± 0.007 | 2.735 ± 0.1 | 0.833 ± 0.03 | 3.568 ± 0.3 | 3.27 ± 0.01 |
| *Nannochloropsis limnetica^#^* | 0.291 ± 0.008 | 0.057 ± 0.003 | 3.285 ± 0.1 | -0.092 ± 0.01 | 3.193 ± 0.02 | -35.86 ± 0.12 |
| *Dunaliella salina* | 0.227 ± 0.007 | 0.075 ± 0.003 | 2.495 ± 0.08 | 0.508 ± 0.02 | 3.003 ± 0.1 | 4.91 ± 0.1 |

**S1 Table.** Original absorbance readings of acetone:DMSO pigment extracts and the calculated Chl *a* and Chl *b* content, total Chl (*a+b*) content and the Chl *a/b* ratio. Equation 1 was used for calculating Chl *a* and Equation 2 was used for calculating Chl *b.*

Chl *a* (µg mL^-1^) = 11.75*A_663_ - 2.35*A_645_ (1)

Chl *b* (µg mL^-1^) = 18.61*A_645_ – 3.96*A_663_ (2)

^#^In *N. limnetica* the calculated Chl *b* content and Chl a/b ratio cannot be accurately interpreted due to the lack of Chl *b.*
