# Supplementary material for: A multi-parametric screening platform for photosynthetic trait characterization of microalgae and cyanobacteria under inorganic carbon limitation
Source: PLoS One. 2020 Jul 23;15(7):e0236188. doi: 10.1371/journal.pone.0236188 (PMC7377499; doi:10.1371/journal.pone.0236188)
Supplement: S4 Fig — Simultaneous measurements of (A) Oxygen evolution and (B) Effective quantum yield of PSII (Y(II)) and (C) pH were performed. Y(II) and pH was measured every 5 min. The cells were kept in the dark for 20 min (grey shade region) then illuminated with actinic white light at 56 μmol photons m-2 s-1. The dots in panel A represent the defined measuring time points of chlorophyll and NADPH fluorescence kinetics at: T0 (control) after 3 min in the dark; T1—initial Ci-replete phase (80 min) in the light; T2—early phase of Ci depletion (170 min); T3—prolonged Ci-depleted phase (300 min); T4—Ci recovery phase (335 min) in the light (arrow indicates the addition of 10 mM NaHCO3). (DOCX) [file pone.0236188.s005.docx]

**S4 Fig. Representative graph of physiological response of *Chlorella sorokiniana* the course of Ci limitation.** Simultaneous measurements of (A) Oxygen evolution and (B) Effective quantum yield of PSII (Y(II)) and (C) pH were performed. Y(II) and pH was measured every 5 min. The cells were kept in the dark for 20 min (grey shade region) then illuminated with actinic white light at 56 µmol photons m^-2^ s^-1^. The dots in panel A represent the defined measuring time points of chlorophyll and NADPH fluorescence kinetics at: T0 (control) after 3 min in the dark; T1 - initial Ci-replete phase (80 min) in the light; T2 - early phase of Ci depletion (170 min); T3 - prolonged Ci-depleted phase (300 min); T4 - Ci recovery phase (335 min) in the light (arrow indicates the addition of 10 mM NaHCO_3_).
