# Supplementary material for: A multi-parametric screening platform for photosynthetic trait characterization of microalgae and cyanobacteria under inorganic carbon limitation
Source: PLoS One. 2020 Jul 23;15(7):e0236188. doi: 10.1371/journal.pone.0236188 (PMC7377499; doi:10.1371/journal.pone.0236188)
Supplement: S7 Fig — Representative traces of the first derivatives of oxygen evolution (O2, black) and ETR (red). The blue arrow represents the time of NaHCO3 addition. (A) Synechocystis sp. PCC 6803 WT grown at 3% CO2, (B) Synechocystis sp. PCC 6803 WT grown at ambient CO2, (C) Synechocystis M55 mutant, (D) Chlorella sorokiniana, (E) Nannochloropsis limnetica, (F) Dunaliella salina (DOCX) [file pone.0236188.s008.docx]

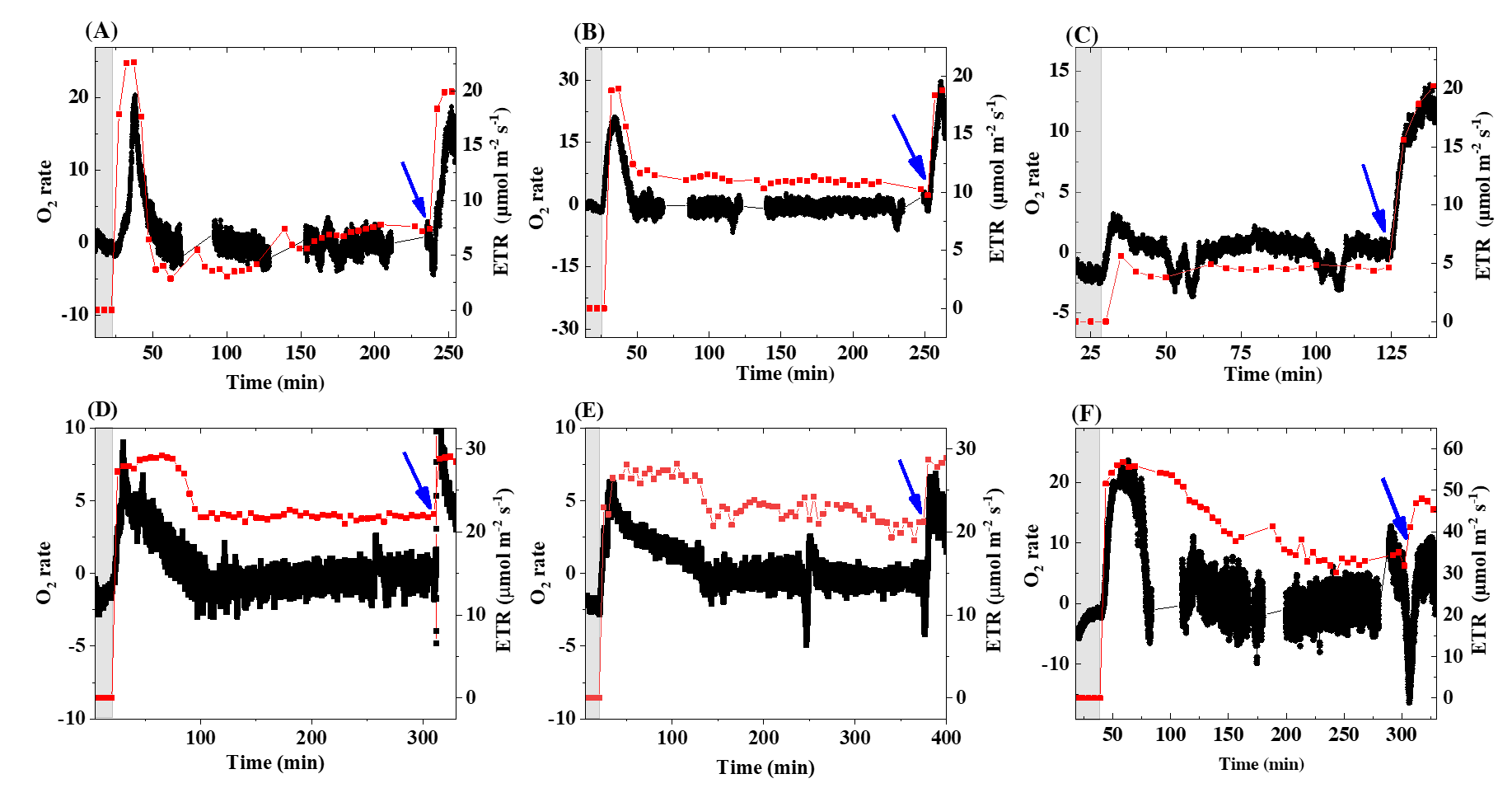


**S7 Fig. Oxygen evolution rate and electron transport rate (ETR) during the course of Ci limitation.** Representative traces of the first derivatives of oxygen evolution (O_2_, black) and ETR (red). The blue arrow represents the time of NaHCO_3_ addition. (A) *Synechocystis* sp. PCC 6803 WT grown at 3% CO_2_, (B) *Synechocystis* sp. PCC 6803 WT grown at ambient CO_2_, (C) *Synechocystis* M55 mutant, (D) *Chlorella sorokiniana*, (E) *Nannochloropsis limnetica*, (F) *Dunaliella salina*
