# Supplementary material for: A multi-parametric screening platform for photosynthetic trait characterization of microalgae and cyanobacteria under inorganic carbon limitation
Source: PLoS One. 2020 Jul 23;15(7):e0236188. doi: 10.1371/journal.pone.0236188 (PMC7377499; doi:10.1371/journal.pone.0236188)
Supplement: S8 Fig — The blue arrow represents the time of NaHCO3 addition. (A) Synechocystis sp. PCC 6803 WT grown at 3% CO2, (B) Synechocystis sp. PCC 6803 WT grown at ambient CO2, (C) Synechocystis M55 mutant, (D) Chlorella sorokiniana, (E) Nannochloropsis limnetica, (F) Dunaliella salina (DOCX) [file pone.0236188.s009.docx]

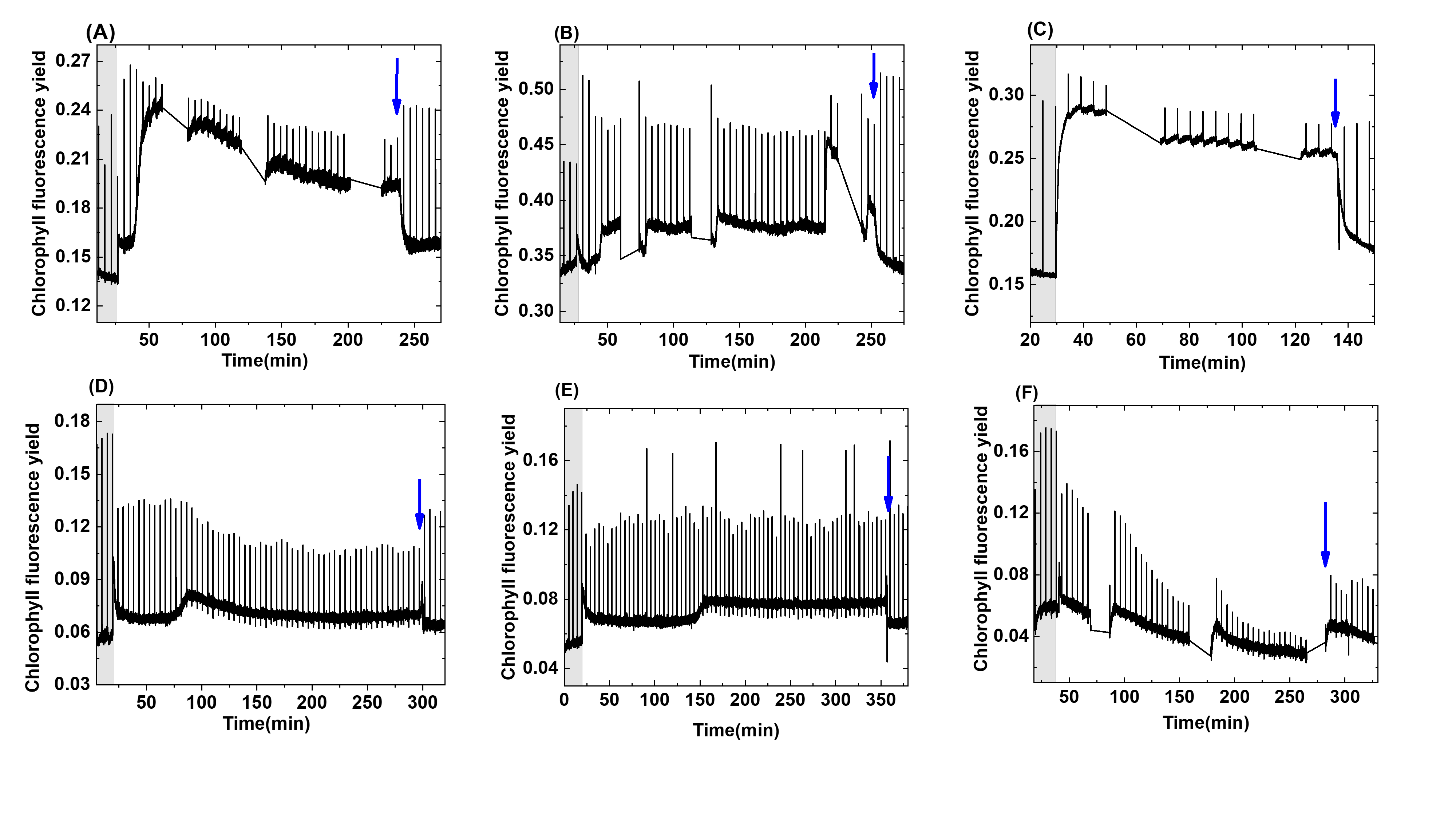


**S8 Fig. Representative chlorophyll fluorescence traces during the course of Ci limitation.** The blue arrow represents the time of NaHCO_3_ addition. (A) *Synechocystis* sp. PCC 6803 WT grown at 3% CO_2_, (B) *Synechocystis* sp. PCC 6803 WT grown at ambient CO_2_, (C) *Synechocystis* M55 mutant, (D) *Chlorella sorokiniana*, (E) *Nannochloropsis limnetica*, (F) *Dunaliella salina*
