# Supplementary material for: A multi-parametric screening platform for photosynthetic trait characterization of microalgae and cyanobacteria under inorganic carbon limitation
Source: PLoS One. 2020 Jul 23;15(7):e0236188. doi: 10.1371/journal.pone.0236188 (PMC7377499; doi:10.1371/journal.pone.0236188)
Supplement: S9 Fig — Representative NADPH fluorescence traces of WT Synechocystis sp. PCC 6803 WT in the absence (black line) or presence (red line) of 10 mM GA. Cells were grown in the presence of 3% CO2 (as described in Materials and Methods) and dark adapted for 5 min before the measurements. (DOCX) [file pone.0236188.s010.docx]

**

**S9 Fig. Representative NADPH fluorescence traces of WT *Synechocystis* sp. PCC 6803 WT** **in the absence (black line) or presence (red line) of 10 mM GA.** Cells were grown in the presence of 3% CO_2_ (as described in Materials and Methods) and dark adapted for 5 min before the measurements.
