# Supplementary material for: A multi-parametric screening platform for photosynthetic trait characterization of microalgae and cyanobacteria under inorganic carbon limitation
Source: PLoS One. 2020 Jul 23;15(7):e0236188. doi: 10.1371/journal.pone.0236188 (PMC7377499; doi:10.1371/journal.pone.0236188)
Supplement: S11 Fig — Representative OJIP traces that were normalized to F0 level (at 50 μs timepoint) at Ci-replete (black), -depleted (red) and recovery (green) stage undergoing Ci limitation. Cells were dark adapted 3min before every measurement. (A) Synechocystis sp. PCC 6803 WT grown at 3% CO2, (B) Synechocystis sp. PCC 6803 WT grown at ambient CO2, (C) Synechocystis M55 mutant, (D) Chlorella sorokiniana, (E) Nannochloropsis limnetica, (F) Dunaliella salina. (DOCX) [file pone.0236188.s012.docx]

**

**S11 Fig. Representative OJIP traces that were normalized to F_0_ level (at 50 μs timepoint) at Ci-replete (black), -depleted (red) and recovery (green) stage undergoing Ci limitation.** Cells were dark adapted 3min before every measurement. (A) *Synechocystis* sp. PCC 6803 WT grown at 3% CO_2_*,* (B) *Synechocystis* sp. PCC 6803 WT grown at ambient CO_2_*,* (C) *Synechocystis* M55 mutant*,* (D) *Chlorella sorokiniana,* (E) *Nannochloropsis limnetica,* (F) *Dunaliella salina.*
